# Drug‐Primed Self‐Assembly of Platinum‐Single‐Atom Nanozyme to Regulate Cellular Redox Homeostasis Against Cancer

**DOI:** 10.1002/advs.202302703

**Published:** 2023-09-11

**Authors:** Li Zhang, Qian Dong, Yumin Hao, Zihan Wang, Wenjuan Dong, Yang Liu, Yueping Dong, Hongpeng Wu, Shaomin Shuang, Chuan Dong, Zhuo Chen, Xiaojuan Gong

**Affiliations:** ^1^ Institute of Environmental Science Shanxi University Taiyuan 030006 China; ^2^ Molecular Science and Biomedicine Laboratory State Key Laboratory of Chemo/Bio‐Sensing and Chemometrics College of Chemistry and Chemical Engineering College of Biology Hunan University Changsha 410082 China; ^3^ School of Chemistry and Chemical Engineering Shanxi University Taiyuan 030006 China; ^4^ State Key Laboratory of Quantum Optics and Quantum Optics Devices Institute of Laser Spectroscopy Shanxi University Taiyuan 030006 China

**Keywords:** carbon dots, cellular homeostasis, platinum, self‐assembly, single‐atom nanozymes

## Abstract

Single‐atom nanozymes (SAzymes) with high catalytic activity exhibit the potential to disequilibrate the reactive oxygen metabolic balance in the tumor microenvironment (TME), which contains several endogenous reductive substances such as glutathione (GSH). Herein, a novel nano‐assembly (CDs@Pt SAs/NCs@DOX) is first constructed using drug‐primed platinum (Pt) single‐atom or nanocluster nanozymes with a Pt loading of 34.8%, which exhibits prominent dual enzymatic activities to mimic peroxidase (POD) and glutathione oxidase (GSHOx). The unique GSHOx‐like activity can efficiently scavenge GSH with a relatively low *K*_m_ (1.04 mm) and high *V*_max_ (7.46 × 10^−6^ m s^−1^), thus avoiding single oxygen (^1^O_2_) depletion. CDs@Pt SAs/NCs@DOX simultaneously demonstrates low‐temperature photothermal therapy and TME‐ or laser‐controlled disassembly and drug release, which can effectively regulate cellular redox homeostasis and achieve high tumor growth inhibition. These outcomes may provide promising strategies for the preparation of Pt SAzymes with multiple activities and variable‐sized nano‐assemblies, allowing for broader applications of SAzymes and nano‐assemblies in the biomedical field.

## Graphical abstract

CDs@Pt SAs/NCs possess POD‐ and GSHOx‐like activities that effectively regulate intracellular redox homeostasis. The dual‐mode responsive depolymerization behavior of CDs@Pt SAs/NCs@DOX and multimodal synergistic antitumor (nanozyme catalytic therapy, low‐temperature PTT, and CT) improves the efficacy and safety of treatment, thereby generating a remarkable therapeutic outcome.

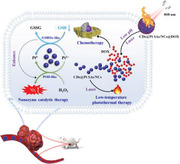

## 1 Introduction

Nanozymes are nanomaterials with enzyme‐like activities and have numerous advantages over most natural enzymes in terms of preparation cost, stability, enzyme activity, and physicochemical properties.^[^ ^1^ ^]^ The single‐atom catalyst (SAC), first reported by Zhang et al.,^[^ ^2^ ^]^ is a promising alternative to conventional enzymes as it can mimic the highly evolved catalytic center of natural enzymes due to its well‐defined electronic and geometric structures.^[^ ^3^ ^]^ Inspired by SACs, single‐atom nanozymes (SAzymes or SAEs) have attracted extensive attention from researchers^[^ ^4^ ^]^ and have been successfully applied in cancer therapy, catalytic degradation of pollutants, biocatalysis, and antibacterial tests.^[^ ^3^, ^4^, ^5^, ^6^, ^7^, ^8^, ^9^, ^10^ ^]^ In the field of tumor therapy, SAzymes can generate variety of reactive oxygen species (ROS), such as single oxygen (^1^O_2_), superoxide radicals (O_2_^−^), hydroxyl radicals (·OH.), peroxides (O_2_^2−^), etc. However, glutathione (GSH), a ROS scavenger, is overexpressed in the tumor microenvironment (TME) (up to 10 mm), and it hampers the efficacy of the ROS‐enhanced cancer treatment. Therefore, efficient control and depletion of GSH in the TME are crucial to improve the efficiency of cancer treatment. Glutathione oxidase (GSHOx) plays an important role in converting GSH to glutathione disulfide (GSSG), which helps in regulating cellular redox homeostasis^[^ ^5^ ^]^; however, reports on this phenomenon are scarce.^[^ ^5^, ^6^, ^8^, ^10^ ^]^ Previous reports have suggested that platinum (Pt) single atoms can consume GSH or enhance the ability of a carrier to consume GSH; however, whether Pt single atoms possess GSHOx‐like activity has not been comprehensively investigated.^[^ ^10^, ^11^, ^12^ ^]^ Moreover, research on elucidating the catalytic reaction mechanism of Pt SAzymes having GSHOx‐like activity is limited owing to the complexity of single‐atom carriers contained in multimetal‐based materials. Therefore, we aimed to find a metal‐free carrier to load Pt single‐atoms and impart them with GSHOx‐like activity to the carrier.

Among all nanomaterials, carbon dots (CDs) have abundant dangling bonds (─OH, ─C═O, and ─NH_2_) and a high surface‐to‐volume ratio, which facilitates the riveting and exposure of active metal sites.^[^ ^6^ ^]^ Furthermore, CDs have good biocompatibility and low cytotoxicity, owing to which they are often used as carriers for the construction of SAzymes and have been applied in biomedical fields.^[^ ^6^, ^13^, ^14^ ^]^ Nevertheless, the ultra‐small size of CDs (<10 nm) subjects them to a fast renal clearance^[^ ^15^ ^]^ rather than the tumor site.^[^ ^13^, ^14^ ^]^ Consequently, a meticulous strategy is required to improve the enhanced permeability and retention (EPR) effect of CDs as Pt SAzymes carriers to achieve tumor delivery.

Generally, small‐sized nanomaterials can be tuned to appropriate sizes via surface modification or functional assembly for EPR effects.^[^ ^16^ ^]^ Agglomeration of nanomaterials by an external force is vital.^[^ ^13^, ^14^ ^]^ Adriamycin (DOX) has a specific self‐agglomeration ability in different solvents that can controllably modulate nanomaterials growth.^[^ ^17^ ^]^ Owing to the agglomeration behavior of DOX, we used it as a driving force to assemble the prepared CDs‐based Pt SAzyme into large nano‐assemblies to achieve the efficient EPR effect and the low‐temperature photothermal therapy (PTT), which not only dramatically enhance delivery in the tumor, but also mild PPT avoids the damage of normal tissues around tumor. Further, the depolymerization behavior induced by TME stimulation can release DOX, which can exploit the chemotherapeutic properties for synergistic tumor treatment.

**Scheme 1:**
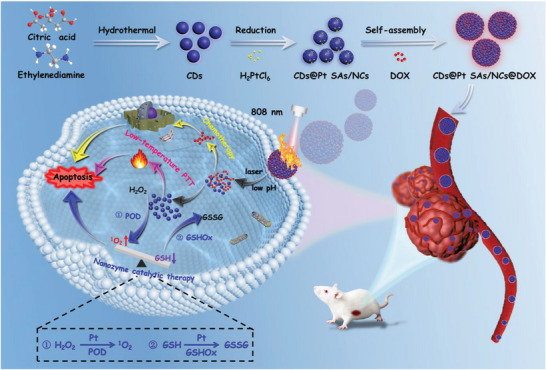
Schematic illustration of the design process and antitumor mechanism of CDs@Pt SAs/NCs@DOX.

Herein, CDs@Pt SAs/NCs were prepared for the first time by reducing Pt single atoms or nanoclusters (Pt SAs/NCs) on CDs. CDs@Pt SAs/NCs have peroxidase (POD)‐ and GSHOx‐like activities that produce large amounts of ^1^O_2_ and deplete excessive GSH. Furthermore, DOX was used as a triggering agent to assemble CDs@Pt SAs/NCs into big‐sized nano‐assemblies (CDs@Pt SAs/NCs@DOX, **Scheme** **1**), and their own large size significantly improves the delivery efficiency. The synergy of nanozyme catalytic therapy, low‐temperature PTT, and chemotherapy (CT) enhance intracellular oxidative stress and regulate intracellular redox homeostasis to achieve antitumor effect. This study introduces a novel concept of Pt SAzymes with multiple enzymes activities and controlled assembly‐disassembly, which might facilitate the application of SAzymes in cancer therapy.

## 2 Results and Discussion

**Figure 1:**
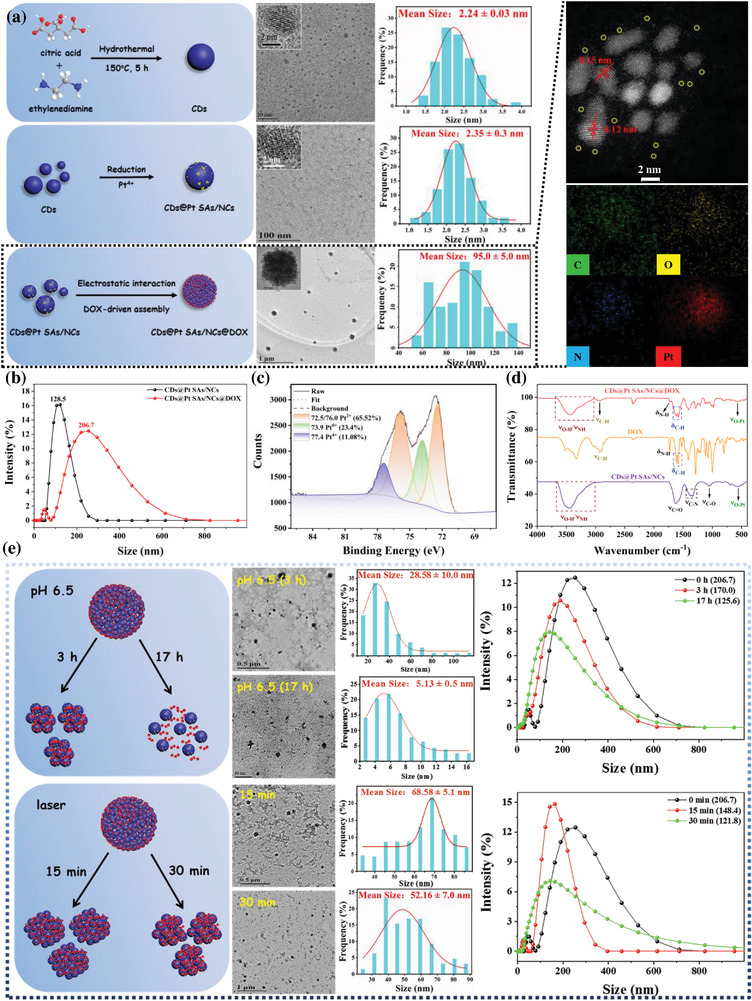
The characterization of CDs@Pt SAs/NCs and CDs@Pt SAs/NCs@DOX. a) The synthesis, TEM image, and particle diameter distribution of CDs, CDs@Pt SAs/NCs, and CDs@Pt SAs/NCs@DOX. Extended diagram: element mapping (top) and HAADF‐STEM (bottom) images of CDs@Pt SAs/NCs@DOX. b) Hydrodynamic diameters of CDs@Pt SAs/NCs and CDs@Pt SAs/NCs@DOX determined by DLS. c) High‐resolution Pt 4f spectrum of CDs@Pt SAs/NCs. d) FT‐IR spectra of CDs@Pt SAs/NCs, DOX, and CDs@Pt SAs/NCs@DOX. e) The changes of TEM, particle size, and hydrodynamic diameter of CDs@Pt SAs/NCs@DOX under different treatment conditions.

The procedures for synthesizing of CDs@Pt SAs/NCs are illustrated in Scheme 1. Transmission electron microscopy (TEM) (**Figure** **1**a), element mapping (Figure S1a, Supporting Information), and high‐angle annular dark‐field scanning transmission electron microscopy (HAADF‐STEM) images (Figure S1b, Supporting Information) confirmed that Pt was successfully reduced on the surface of CDs as single atoms and nanoclusters^[^ ^18^, ^19^ ^]^ to form monodisperse CDs@Pt SAs/NCs with an average diameter of 2.35 ± 0.3 nm; the diameter of CDs@Pt SAs/NCs obtained using dynamic light scattering (DLS) was 128.5 nm (Figure 1b). The considerable differences between the size of CDs@Pt SAs/NCs obtained using DLS and TEM may be attributed to the formation of hydration layers on their surfaces or the inevitable weak aggregation caused by surface functional groups (‐COOH and ‐NH_2_).^[^ ^14^, ^20^, ^21^ ^]^ The chemical composition and surface functional groups of CDs@Pt SAs/NCs were explored using X‐ray photoelectron spectroscopy (XPS, Figure 1c; Figure S2, Supporting Information) and Fourier transform infrared (FT‐IR) spectra (Figure 1d).^[^ ^22^, ^23^, ^24^ ^]^ Inductively coupled plasma optical emission spectrometry (ICP‐OES) revealed the Pt content to be 34.8%. The thermogravimetric analysis (TGA, Figure S3, Supporting Information) result verified the ICP‐OES result and demonstrated the outstanding thermostability of CDs@Pt SAs/NCs. The optical performance of CDs@Pt SAs/NCs was investigated via UV–vis absorption spectra, photoluminescence (PL) spectra, and fluorescence quantum yield (FQY) (Figure S4, Supporting Information), and it was confirmed that Pt SAs/NCs had been successfully reduced to the surface of CDs,^[^ ^25^ ^]^ resulting in the fabrication of CDs@Pt SAs/NCs with excellent performance.

Considering the self‐aggregation behavior of DOX, it was used as a traction agent for the first time in this study to drive the self‐assembly of CDs@Pt SAs/NCs to form a spherical nano‐assembly (CDs@Pt SAs/NCs@DOX), enhancing the EPR effect and introducing CT. CDs@Pt SAs/NCs@DOX was synthesized via the self‐aggregation of DOX and electrostatic adsorption between CDs@Pt SAs/NCs and DOX with a DOX loading efficiency of 13.38%. The size of CDs@Pt SAs/NCs@DOX (95.0 ± 5.0 nm) was larger than that of CDs@Pt SAs/NCs (2.35 ± 0.3 nm), implying that the addition of DOX led to the aggregation of CDs@Pt SAs/NCs (Figure 1a). Accordingly, adding DOX increased the hydrodynamic diameter of CDs@Pt SAs/NCs from 128.5 to 206.7 nm (Figure 1b), which is the optimal size for nanomaterials to exert an efficient EPR effect.^[^ ^13^, ^14^, ^16^ ^]^ Element mapping and HAADF‐STEM images of CDs@Pt SAs/NCs@DOX (extended diagram of Figure 1a) show that the nano‐assembly behavior of DOX does not affect the structure of CDs@Pt SAs/NCs. Further, FT‐IR (Figure 1d), zeta potential (Figure S5a, Supporting Information), optical properties (Figure S5b,c, Supporting Information), and TGA curve (Figure S5d, Supporting Information) indicated the successful synthesis of CDs@Pt SAs/NCs@DOX. Moreover, the DOX release behavior of CDs@Pt SAs/NCs@DOX was explored in detail (Figure 1e; Figure S6, Supporting Information). CDs@Pt SAs/NCs@DOX were treated with pH 6.5 for 0, 3, and 17 h with particle size and hydrodynamic diameter of 95.0 and 206.7 nm, 28.58 and 170.0 nm, and 5.13 and 125.6 nm, respectively, demonstrating that DOX, like an important key, can control the aggregation and depolymerization of CDs@Pt SAs/NCs@DOX nano‐assembly (Figure 1e). After the 808 nm laser irradiation for 0, 15, and 30 min, the particle size and hydrodynamic diameter of CDs@Pt SAs/NCs@DOX were 95.0 and 206.7 nm, 68.58 and 148.4 nm, and 52.16 and 121.8 nm, respectively, suggesting that laser irradiation can promote rapid depolymerization of CDs@Pt SAs/NCs@DOX (Figure 1e). We speculated that the binding between CDs@Pt SAs/NCs and DOX relies on electrostatic and hydrogen bonding interactions,^[^ ^26^, ^27^ ^]^ which may be attributed to the formation of a π–π stacking between the phenyl ring of DOX and the sp^2^ structural domain of CDs@Pt SAs/NCs. Besides, the solubility of DOX under acidic conditions was significantly stronger than its solubility under physiological conditions, which contributed to the rapid release of DOX from CDs@Pt SAs/NCs@DOX.^[^ ^27^, ^28^ ^]^ The above results indicate that DOX in CDs@Pt SAs/NCs@DOX was sensitive to the TME (weak acidity, pH 6.5–7.0) and laser irradiation, which can depolymerize CDs@Pt SAs/NCs@DOX and promote a sustainable release of DOX. CDs@Pt SAs/NCs@DOX with this feature has a unique advantage in inhibiting weakly acidic tumor growth.

**Figure 2:**
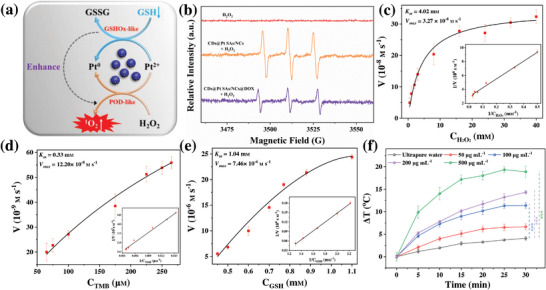
The enzyme‐like activities and photothermal effect of CDs@Pt SAs/NCs. a) Mechanistic diagram of enzyme‐like activities of CDs@Pt SAs/NCs. b) ESR spectra of 2,2,6,6‐tetramethylpiperidine (TEMP)/^1^O_2_ adducts in different solutions. Michaelis–Menten curve for CDs@Pt SAs/NCs and c) H_2_O_2_ or d) TMB. Double‐reciprocal Lineweaver‐Burk plots of CDs@Pt SAs/NCs and H_2_O_2_ (inset of Figure 2c) or TMB (inset of Figure 2d). e) Michaelis–Menten curve for CDs@Pt SAs/NCs and GSH. Inset: Double‐reciprocal Lineweaver–Burk plots of CDs@Pt SAs/NCs and GSH. f) Photothermal conversions of CDs@Pt SAs/NCs with various concentrations under 808 nm laser (0.37 W cm^−2^) (^***^ *p* < 0.001, *n* = 3).

**Figure 3:**
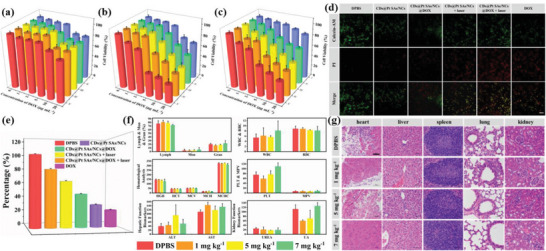
In vitro cytotoxicity and biological safety evaluation of CDs@Pt SAs/NCs@DOX. MTT analysis results on a) LO2, b) HeLa, and c) 4T1 cells (*n* = 6). I: CDs@Pt SAs/NCs, II: CDs@Pt SAs/NCs@DOX, III: CDs@Pt SAs/NCs + laser, IV: CDs@Pt SAs/NCs@DOX + laser, V: DOX. d) Live‐dead staining assay of HeLa cells. Scale bar: 200 µm. e) The quantitative analysis results of living cells in Figure 3d. f) Analysis results of hematological indexes (Lymph%; Mon%; Gran%; WBC, 10^10^ L^−1^; RBC, 10^12^ L^−1^; HGB, g L^−1^; HCT, %; MCV, fL; MCH, pg; MCHC, g L^−1^; PLT, 10^10^ L^−1^; MPV, fL), and blood biochemical indexes (ALT, U L^−1^; AST, U L^−1^; UREA, mm; UA, µM) of the mice after different treatments (*n* = 3). g) H&E‐stained slices of the main organ after different treatments. Scale bar: 50 µm.

The enzyme activity of CDs@Pt SAs/NCs was comprehensively studied (**Figure** **2**a). UV–vis absorption spectra revealed that CDs@Pt SAs/NCs exhibit excellent POD‐like activity and perform the factor‐dependent (Figure S7a–e, Supporting Information). The characteristic ^1^O_2_ (1:1:1) signal can be clearly observed in the electron spin resonance (ESR) spectra of CDs@Pt SAs/NCs solutions (Figure 2b), while the characteristic signal of ·OH (1:2:2:1) cannot be detected (Figure S7f, Supporting Information).^[^ ^5^, ^6^ ^,^ ^29^ ^]^ Additionally, Michaelis–Menten‐like curves of CDs@Pt SAs/NCs were achieved using H_2_O_2_ (Figure 2c) and 3,3′,5,5′‐tetramethylbenzidine (TMB, Figure 2d) as substrates. Compared with natural horseradish peroxidase (HRP) and other SAzymes (Table S1, Supporting Information), CDs@Pt SAs/NCs had a lower *K*_m_ and a higher *V*_max_, suggesting that CDs@Pt SAs/NCs have a strong affinity for the substrate and high catalytic reaction rate. As the generated ^1^O_2_ may be consumed by the overproduced GSH in the TME, we investigated whether CDs@Pt SAs/NCs have GSHOx‐like activity. Studying the PL and UV–vis absorption spectra of CDs@Pt SAs/NCs in the presence of GSH revealed that CDs@Pt SAs/NCs can deplete GSH (Figure S8a–c, Supporting Information). After CDs@Pt SAs/NCs react with GSH, a new S 2p peak and S‐S were identified in the XPS survey scan (Figure S8d, Supporting Information) and high‐resolution S 2p spectrum (Figure S8e, Supporting Information), respectively; this observation confirmed that GSH can be converted to GSSG. The Pt^0+^ content in CDs@Pt SAs/NCs increased from 23.4% (Figure 1c) to 41.24% (Figure S8f, Supporting Information) after CDs@Pt SAs/NCs react with GSH, indicating that GSH can reduce Pt^2+^ to Pt^0+^. We speculate that the shedding of a small fraction of Pt^0+^ exposed some nitrogen atoms present on the surface of CDs@Pt SAs/NCs, thereby changing the FQY of CDs@Pt SAs/NCs after the addition of GSH from 3.05% (Figure S4g–h, Supporting Information) to 5.8% (Figure S8g–h, Supporting Information), which is much lower than the FQY of CDs (Figure S4e‐f, Supporting Information). Therefore, it can be suggested that most Pt SAs/NCs are retained on the surface of CDs. Meanwhile, CDs@Pt SAs/NCs exhibited Michaelis–Menton‐like kinetics in the catalytic reaction of GSH (Figure 2e) with *K*_m_ and *V*_max_ of 1.04 mm and 7.46 × 10^−6^ m s^−1^, respectively, which were comparable to the *K*_m_ and *V*_max_ reported for the GSHOx‐like nanozymes (Table S2, Supporting Information). These results illustrate that CDs@Pt SAs/NCs have POD‐ and GSHOx‐like activities, which can consume GSH with an atomic economy to avoid the consumption of the generated ^1^O_2_ and result in amplifying the efficacy of antitumor therapy. Notably, this is the first report demonstrating that Pt‐based nanozymes can mimic POD and GSHOx activities with high catalytic efficiency. CDs@Pt SAs/NCs@DOX exhibited the same perfect POD‐like activity as CDs@Pt SAs/NCs and produced ^1^O_2_ (Figure 2b; Figure S9a–g and Table S1, Supporting Information). The PL intensity of CDs@Pt SAs/NCs@DOX gradually enhanced with the increase of GSH, illustrating that CDs@Pt SAs/NCs@DOX has similar GSHOx‐like activity as CDs@Pt SAs/NCs (Figure S9h, Supporting Information).

The feasibility of using CDs@Pt SAs/NCs as a photothermal converter was further studied because CDs@Pt SAs/NCs have a broad absorption spectrum with strong absorption in the range of 500–900 nm (Figure S4a, Supporting Information). The temperature of CDs@Pt SAs/NCs gradually rose with the increase of CDs@Pt SAs/NCs concentration or irradiation time (Figure 2f), suggesting the photothermal conversion effect of CDs@Pt SAs/NCs is related to concentration and time. The temperature of CDs@Pt SAs/NCs remained stable after six cycles of laser irradiation (Figure S10a, Supporting Information), which indicated that CDs@Pt SAs/NCs have excellent photostability. The photothermal conversion efficiency (*η*) of CDs@Pt SAs/NCs was ≈7.73% (Figure S10b, Supporting Information).^[^ ^30^ ^]^ Although the *η* value of CDs@Pt SAs/NCs is lower than that of other materials,^[^ ^31^, ^32^ ^]^ a low photothermal treatment (temperature ≤ 45 °C) can be performed on tumors, effectively preventing damage to normal cells.^[^ ^33^ ^]^ CDs@Pt SAs/NCs@DOX have the same photothermal properties as CDs@Pt SAs/NCs (Figure S10c–f, Supporting Information). In particular, the higher *η* value of CDs@Pt SAs/NCs@DOX (10.38%) may be attributed to its larger size and more stable polymerization state than those of CDs@Pt SAs/NCs. These results confirmed that CDs@Pt SAs/NCs@DOX perfectly inherited the enzyme‐like activities of CDs@Pt SAs/NCs and can avoid damage to normal tissues around the tumor.

The classical methyl thiazolyl tetrazolium (MTT) assays on the human normal liver (LO2), human cervical cancer (HeLa), and murine mammary carcinoma (4T1) cells were performed to verify in vitro cytotoxicity (**Figure** **3**a–c). Compared with LO2 cells, the survival rates of HeLa and 4T1 cells gradually decreased with the increasing concentration of CDs@Pt SAs/NCs, indicating that CDs@Pt SAs/NCs have favorable enzyme‐like activity in cancer cells. At the same concentration of CDs@Pt SAs/NCs and CDs@Pt SAs/NCs@DOX, the survival rates of HeLa and 4T1 cells substantially decreased after laser irradiation, whereas the survival rate of LO2 cells did not change. This observation indicated that cancer cells and normal cells have different intakes of CDs@Pt SAs/NCs or CDs@Pt SAs/NCs@DOX. When used in similar concentrations for HeLa and 4T1 cells, CDs@Pt SAs/NCs@DOX had higher cytotoxicity than CDs@Pt SAs/NCs due to the release of DOX from CDs@Pt SAs/NCs@DOX in the TME that can inhibit the growth of cancer cells. CDs@Pt SAs/NCs@DOX combined with laser displayed the strongest inhibitory effect on HeLa and 4T1 cells, which strongly suggests that CDs@Pt SAs/NCs@DOX can synergically exert enzyme‐like, photothermal, and chemotherapeutic activities to inhibit the growth of cancer cells. Besides, similar cytotoxicity results can be further proven by performing live‐dead staining analysis (Figure 3d) and calculating the percentage of viable cells (Figure 3e). Furthermore, the uptake ability of HeLa cells to different concentrations of CDs@Pt SAs/NC and CDs@Pt SAs/NCs@DOX was investigated in detail. As depicted in Figure S11 (Supporting Information), the Pt content in CDs@Pt SAs/NCs@DOX‐incubated HeLa cells was much higher than that in CDs@Pt SAs/NCs‐incubated HeLa cells, suggesting that CDs@Pt SAs/NCs@DOX can be more efficiently uptaken by HeLa cells, which may be attributed to the fact that CDs@Pt SAs/NCs@DOX had a larger size^[^ ^34^, ^35^ ^]^ and the release of DOX affected the cellular state,^[^ ^36^ ^]^ facilitating the cellular uptake of CDs@Pt SAs/NCs@DOX.

Subsequently, the biocompatibility of CDs@Pt SAs/NCs@DOX was extensively evaluated in healthy mice. It was found that the hematological parameters of the treated mice were within the standard range (Figure 3f), and the body weight of the mice displayed reasonable fluctuation during the treatments (Figure S12, Supporting Information), revealing the outstanding biosafety and biocompatibility of CDs@Pt SAs/NCs@DOX. Hepatic (alanine transaminase [ALT] and aspartate aminotransferase [AST]) and kidney (urea [UREA] and uric acid [UA]) function biomarkers did not exhibit remarkable changes compared to the control group (Figure 3f), showing that CDs@Pt SAs/NCs@DOX had no apparent hepatotoxicity and nephrotoxicity. The major excised organs of the mice were analyzed by hematoxylin and eosin (H&E) staining. No obvious pathological changes were found in the slice of major organs after intravenous injection of CDs@Pt SAs/NCs@DOX in mice (Figure 3g), confirming the minimal in vivo systemic toxicity of CDs@Pt SAs/NCs@DOX. Additionally, the optimal concentration of CDs@Pt SAs/NCs@DOX for antitumor therapy in mice was found to be 5 mg kg^−1^.

**Figure 4:**
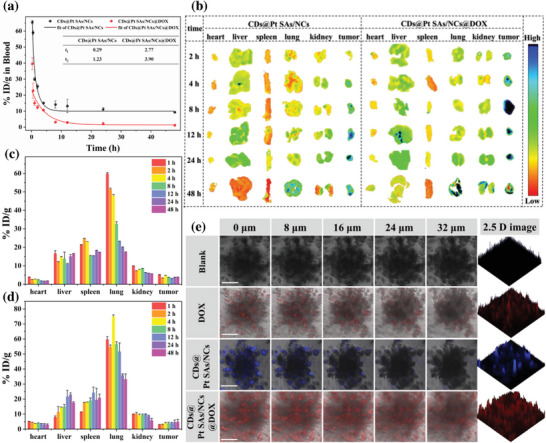
Pharmacokinetics, biodistribution, and tissue‐penetration behavior of CDs@Pt SAs/NCs@DOX. a) Blood clearance profile of CDs@Pt SAs/NCs and CDs@Pt SAs/NCs@DOX in 4T1 tumor‐bearing mice. b) Ex vivo fluorescence imaging of the excised tumor and main organs after different treatments in 4T1 tumor‐bearing mice. The fluorescent signals were collected from CDs in CDs@Pt SAs/NCs and CDs@Pt SAs/NCs@DOX. Biodistribution of c) CDs@Pt SAs/NCs@DOX and d) CDs@Pt SAs/NCs in major organs and tumor tissues at different times of post‐injection (*n* = 3). e) LSCM and 2.5 D images of 4T1 MCSs after different treatments. Scale bar: 50 µm.

The pharmacokinetics behaviors of CDs@Pt SAs/NCs and CDs@Pt SAs/NCs@DOX were investigated in detail. The circulation of CDs@Pt SAs/NCs and CDs@Pt SAs/NCs@DOX in blood conformed to a two‐compartment pharmacokinetic model with 0.29 and 1.23 h and 2.77 and 3.90 h for the first and second phases, respectively (**Figure** **4**a). The high circulatory half‐life of CDs@Pt SAs/NCs@DOX may be due to their large size that ensures an efficient EPR effect. The extended blood circulation time suggested that CDs@Pt SAs/NCs@DOX can evade recognition and elimination by the reticuloendothelial system.^[^ ^37^ ^]^

The biodistribution of CDs@Pt SAs/NCs@DOX was investigated in 4T1 tumor‐bearing BALB/c mice. The fluorescence of CDs@Pt SAs/NCs@DOX in the tumor was weak at 4 h post‐injection and the fluorescence of the tumor was remarkably enhanced at 8 h post‐injection, whereas CDs@Pt SAs/NCs began to be enriched at the tumor site at 8 h post‐injection (Figure 4b). The above results revealed that CDs@Pt SAs/NCs@DOX accumulated much faster than CDs@Pt SAs/NCs in tumors, which may be attributed to the efficient EPR effect of CDs@Pt SAs/NCs@DOX. Meanwhile, at 48 h post‐injection, the fluorescence of tumors in CDs@Pt SAs/NCs@DOX‐treated mice was stronger than that in CDs@Pt SAs/NCs‐treated mice, suggesting a slower in vivo blood clearance of CDs@Pt SAs/NCs@DOX. At all the tested time points, the fluorescence of tumors in CDs@Pt SAs/NCs@DOX‐treated mice was significantly higher than that of all organs. Since the self‐aggregation of DOX can lead to fluorescence quenching, we believe that the above phenomenon is caused by the polymerization of CDs@Pt SAs/NCs@DOX at the tumor. The contents of Pt in the major organs and tumors were further calculated using ICP‐OES to determine the distribution of CDs@Pt SAs/NCs@DOX and CDs@Pt SAs/NCs in mice (Figure 4c,d). Similar to several nanomaterials that have been reported for their use in biomedicine, CDs@Pt SAs/NCs@DOX can be captured by the pulmonary reticuloendothelial system at 48 h post‐injection.^[^ ^15^ ^]^ As ex vivo fluorescence imaging and ICP‐OES are two completely different analysis methods, it is normal that there are some differences in the obtained results; however, the conclusions drawn from these tests are essentially the same. Therefore, it can be concluded that CDs@Pt SAs/NCs@DOX is a nano‐assembly with excellent performance and can be used for antitumor therapy (vide infra).

The internalization behavior and tissue penetration of CDs@Pt SAs/NCs@DOX were further investigated in 4T1 multicellular spheroids (MCSs) (Figure 4e). CDs@Pt SAs/NCs@DOX‐treated 4T1 MCSs displayed intense red fluorescence signals in deep layers and core, whereas DOX‐ or CDs@Pt SAs/NCs‐treated 4T1 MCSs exhibited fluorescence signals only in the superficial layers, thereby demonstrating that CDs@Pt SAs/NCs@DOX can penetrate deeper tissues and has significant therapeutic potential in solid tumors.

**Figure 5:**
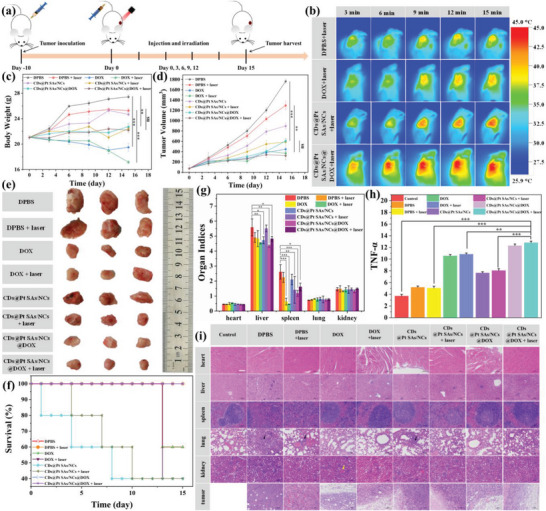
In vivo tumor suppression study. a) Schematic illustration of the treatment protocol. b) Thermographic images of 4T1 tumor‐bearing mice with different treatments. c) The body weight and d) tumor volume of the mice in all groups during the treatment for 15 days. e) Representative digital photos of excised tumors in all treatment groups. f) Survival rate of the mice in all treatment groups during 15 days. g) Comparison of the organ indices of the mice after 25 days of inoculation. h) TNF‐α level of mice after various treatments. i) H&E staining of main organs and tumors after different treatments (Scale bar: 100 µm). (^*^ *p* < 0.05, ^**^ *p* < 0.01, ^***^ *p* < 0.001, *ns*: not significant).

The results of in vitro experiments suggested that CDs@Pt SAs/NCs@DOX has great potential for in vivo tumor therapy. 4T1 tumor‐bearing mice were generated 10 days post‐4T1 cell inoculation and randomly divided into eight groups. They were treated with (G1) Dulbecco's phosphate‐buffered saline (DPBS), (G2) DPBS + laser, (G3) DOX, (G4) DOX + laser, (G5) CDs@Pt SAs/NCs, (G6) CDs@Pt SAs/NCs + laser, (G7) CDs@Pt SAs/NCs@DOX, and (G8) CDs@Pt SAs/NCs@DOX + laser on days 0, 3, 6, 9, and 12 for five times (**Figure** **5**a). Completely healthy mice without any treatment served as a blank control group. After 15 min of laser irradiation, the temperature of the tumor site was much higher in the mice treated with CDs@Pt SAs/NCs + laser or CDs@Pt SAs/NCs@DOX + laser (G6 and G8 groups) than in the mice treated with DPBS + laser or DOX + laser (G2 and G4 groups) (Figure 5b). The perfect EPR effect of CDs@Pt SAs/NCs@DOX induced a maximum temperature of only 45.5 °C in the mice treated with CDs@Pt SAs/NCs@DOX + laser (G8 group), which basically falls under the category of mild heat treatment^[^ ^33^ ^]^ and does not easily cause an inflammatory response. The body weight of mice in the free DOX‐treated groups (G3 and G4 groups) decreased significantly, whereas the body weight of mice in the CDs@Pt SAs/NCs@DOX‐treated groups (G7 and G8 groups) increased with prolongation of the treatment time (Figure 5c), implying that multiple injections of CDs@Pt SAs/NCs@DOX did not cause any noticeable side effects. This result was confirmed by the representative photographs of the mice with tumors (Figure S13, Supporting Information). Further, the tumor volume (Figure 5d), tumor volume change (Figure S14a, Supporting Information), and tumor growth inhibition (TGI, Figure S14b, Supporting Information) were explored. The results indicated that DOX, CDs@Pt SAs/NCs, and CDs@Pt SAs/NCs@DOX exhibited different degrees of TGI compared with the DPBS group (G1 group). These comparative results demonstrated that the best tumor suppression effect was seen in the CDs@Pt SAs/NCs@DOX + laser‐treated mice (G8 group) owing to the triple action of nanozyme catalytic therapy, low‐temperature PTT, and chemotherapy. The TGI of CDs@Pt SAs/NCs@DOX was higher than that of CDs@Pt SAs/NCs, which may be related to its efficient EPR effect and the presence of DOX. This result was further verified by examining the typically excised tumors after the treatments (Figure 5e). The lower survival rate in the DOX‐ and CDs@Pt SAs/NCs‐treated mice (G3‐G6 groups) implied that the low enrichment of DOX and CDs@Pt SAs/NCs at the tumor site induced side effects (Figure 5f). In contrast, the CDs@Pt SAs/NCs@DOX‐treated mice (G7 and G8 groups) revealed 100% survival rate, establishing that CDs@Pt SAs/NCs@DOX prolonged the survival rate of tumor‐bearing mice and considerably reduced the side effects of DOX and CDs@Pt SAs/NCs. Organ indices can also provide important information on the systemic toxicity of nanomaterials.^[^ ^38^ ^]^ No significant differences in heart, lung, and kidney indices of the control (G1 and G2 groups) and experimental (G2–G8 groups) groups were found (Figure 5g), indicating that multiple injections do not cause prominent toxicity and side effects. Although the experimental results depicted in Figure 4c indicate that CDs@Pt SAs/NCs@DOX are readily captured by the reticular structure of the lung, they cause little damage to lung and little pulmonary toxicity. Compared with the CDs@Pt SAs/NCs‐ and CDs@Pt SAs/NCs@DOX‐treated mice (G5 and G7 groups), the liver and spleen of DOX‐treated mice (G3 and G4 groups) displayed obvious atrophy and low function,^[^ ^38^ ^]^ illustrating that DOX has noticeable hepatotoxicity and splenic toxicity. For the CDs@Pt SAs/NCs@DOX + laser‐treated mice (G8 group), the effects of CDs@Pt SAs/NCs@DOX on the liver and spleen were within acceptable limits. In addition, no abnormal clinical symptoms or behaviors, such as anorexia, malaise, or death, were observed throughout the study in CDs@Pt SAs/NCs@DOX‐treated mice (G7 and G8 groups). These results validate that CDs@Pt SAs/NCs@DOX can significantly reduce in vivo toxicity caused by naked DOX, but does not affect the therapeutic efficacy of DOX. Hematological and blood biochemical assays were further performed to assess blood cells and major organ function (Figure S15, Supporting Information). Except for Lymph%, Gran%, and WBC, the other hematological parameters of all mice fluctuated within the standard range without significant changes (Figure S15a, Supporting Information). The Lymph% and WBC of the DOX‐treated mice (G3 and G4 groups) were lower than those in the CDs@Pt SAs/NCs@DOX‐treated mice (G7 and G8 groups), indicating that DOX caused damage to the hematopoietic stem cells and induced myelosuppression in mice. The DOX‐treated mice (G3 and G4 groups) had higher Gran% than the CDs@Pt SAs/NCs@DOX‐treated mice (G7 and G8 groups) due to DOX‐induced infection or hemolysis. These results suggest that CDs@Pt SAs/NCs@DOX not only prevent the risk of free DOX‐induced myelosuppression but the introduction of laser can improve its therapeutic efficacy without inducing side effects. ALT and AST levels were significantly higher in DOX‐treated mice than in CDs@Pt SAs/NCs@DOX + laser‐treated mice and control mice, indicating that DOX caused damage to the hepatocytes and tissue in mice (Figure S15b, Supporting Information). The levels of renal function biomarkers, including UREA and UA, were similar in the experimental and control mice (Figure S15c, Supporting Information), demonstrating that CDs@Pt SAs/NCs@DOX had no obvious nephrotoxicity. The TNF‐α levels in DOX‐ or CDs@Pt SAs/NCs@DOX‐treated mice were significantly higher than those in the other groups (Figure 5h), indicating that DOX and CDs@Pt SAs/NCs@DOX induced a strong immune response in mice. H&E staining of the tumor and major organs was performed to evaluate the organ toxicity and treatment effects (Figure 5i). The major organ slices of CDs@Pt SAs/NCs@DOX‐treated mice were normal and did not exhibit obvious pathological changes, testifying that CDs@Pt SAs/NCs@DOX had minimal cardiotoxicity, hepatotoxicity, splenic toxicity, pulmonary toxicity, and nephrotoxicity in mice. Few tumor cells were observed in the lungs of the control and CDs@Pt SAs/NCs‐treated mice (G1, G2, and G5 groups), which was probably due to 4T1 cell metastasis.^[^ ^39^ ^]^ Some focal hemorrhages were observed in the kidneys of the DOX‐treated mice, implying that DOX may have some nephrotoxicity. Except for the control groups (G1 and G2 groups), the tumor slices of the other groups exhibited inflammatory cell infiltration and varying degrees of tumor tissue damage, including irregular cell morphology, cytokinesis, and lysis of tumor cell nuclei. The apoptosis and necrosis rates of tumor slices in the CDs@Pt SAs/NCs@DOX‐treated mice (G8 group) were higher than the other groups, indicating that CDs@Pt SAs/NCs@DOX had good antitumor effects. Besides, no detectable metastatic lesions were found in the major organs of the CDs@Pt SAs/NCs@DOX‐treated mice (G7 and G8 groups), demonstrating the feasibility of CDs@Pt SAs/NCs@DOX against metastasis. These findings confirm the great potential of CDs@Pt SAs/NCs@DOX for triple therapy of tumors combining nanozyme catalytic therapy, low‐temperature PTT, and CT.

## 3 Conclusion

For the first time, we have developed a DOX‐primed CDs‐based Pt SAs/NCs super‐stable nano‐assembly (CDs@Pt SAs/NCs@DOX) for regulating cellular redox homeostasis and combining nanozyme catalytic therapy, low‐temperature PTT, and CT against malignant tumors. Compared with the other reported nano‐systems for tumor treatment, our nano‐assembly offers several advantages: 1) CDs@Pt SAs/NCs@DOX can generate ^1^O_2_ and consume GSH via biochemical reactions occurring in the TME, thereby amplifying the oxidative stress response in tumors. 2) The DOX‐driven formation of CDs@Pt SAs/NCs@DOX improved their enrichment efficiency in tumors and prolonged their clearance time in the blood. 3) The synergistic treatment of CDs@Pt SAs/NCs@DOX against tumor sites ensures biosafety and improves the efficiency of tumor treatment. In summary, we used CDs as carriers to self‐assemble Pt SAzymes using the driving behavior of DOX for constructing CDs@Pt SAs/NCs@DOX with biomimetic enzymatic activity, expanding the application of CDs and SAzymes in bio‐nanomedicine.

## 4 Experimental Section

### Materials

Citric acid, adriamycin, 5,5′‐Dithiobis‐(2‐nitrobenzoic acid) (DTNB), 3,3′,5,5′‐Tetramethylbenzidine (TMB), Dulbecco's phosphate‐buffered saline (DPBS), Calcein‐AM, GSH, 5,5‐dimethyl‐1‐pyrroline‐*N*‐oxide (DMPO), and 2,2,6,6‐tetramethylpiperidine (TEMP) were acquired from Aladdin Biochemical Technology Co., Ltd. (Shanghai, China). Propidium iodide (PI) was purchased from Solarbio Science & Technology Co., Ltd. (Beijing, China). Chloroplatinic acid hexahydrate was obtained from Macklin Biochemical Co., Ltd. (Shanghai, China). Dulbecco's modified eagle medium (DMEM), fetal bovine serum (FBS), trypsin, penicillin, streptomycin, and methyl thiazolyl tetrazolium (MTT) were acquired from Sangon Biological Engineering Co., Ltd. (Shanghai, China). Nitric acid (HNO_3_) and hydrochloric acid (HCl) were gotten from Fuyu Fine Chemical Co., Ltd. (Tianjin, China). All purchased reagents were analytically pure and used directly without any processing. Ultrapure water was prepared by a Molecular Ultrapure Water System (Shanghai, China) with a specific resistance of 18.25 MΩ cm.

### Characterizations

The transmission electron microscopic (TEM) image, the element mapping images, and the high‐resolution transmission electron microscopic (HRTEM) image were gained on a JEOL JEM‐2100 transmission electron microscopy (Tokyo, Japan). High‐angle annular dark‐field scanning transmission electron microscopy (HAADF‐STEM) images were taken on a 3.2 Themis Z electron microcopy (Thermo Scientific, Holland). The X‐ray photoelectron spectra (XPS) and Fourier transform infrared spectra (FT‐IR) were carried out on an AXIS ULTRA DLD X‐ray photoelectron spectrometer (Kratos, Tokyo, Japan) and a Bruker Tensor II FTIR spectrometer (Bremen, Germany), respectively. The UV–vis absorption and photoluminescence (PL) spectra were performed on a Lambda 365 UV/vis absorption spectrophotometer (PerkinElmer, Llantrisant, UK) at 200–800 nm or a Lambda 950 spectrophotometer (PerkinElmer, Llantrisant) and a Varian Cary Eclipse spectrofluorometer (Palo Alto, CA, USA), respectively. Inductively coupled plasma optical emission spectrometry (ICP‐OES) measurements were carried out via Optical Emission Spectrometer (PerkinElmer, Avio 500, USA). Photothermal effect was evaluated under the laser irradiation of 808 nm (Changchun New Industries Optoelectronics Technology Co., Ltd., China). Zeta potential and dynamic light scattering (DLS) were from a Zetasizer Nano ZS90 (Malvern, Worcestershire, UK). ESR spectra were measured at EMXPLUS10/12 (Bruker, USA). The infrared thermal images and temperature changes of the tumor site were recorded by 869 Testo Thermal Imagers (Testo Instrument international trading Co., Ltd., Shanghai, China). Cells images and 4T1 multicellular spheroids images were captured on an LSM‐710 airy scan super‐resolution confocal microscope (Zeiss, German). The organ's fluorescence imaging was conducted by Bruker's In Vivo Fx Pro living imaging system (Bruker, USA).

### Synthesis of CDs@Pt SAs/NCs

After citric acid (1.0507 g) and ethylenediamine (335 µL) were completely dissolved in ultrapure water (10 mL), they were transferred into a Teflon‐line stainless steel autoclave and heated at 150 °C for 5 h to obtain the brown solution. The obtained solution was dialyzed in a dialysis bag (1000 Da) against ultrapure water for 24 h to remove the unreacted raw materials, which was further lyophilized to obtain brown CDs solid powder. Under magnetic stirring, H_2_PtCl_6_ (5 mL, 37.3 mm) was dropwise added into CDs (5 mL, 1.0 mg mL^−1^), and then NaOH (0.5 m) was used to adjust the pH of the solution to 12.0. Excess NaBH_4_ (10 mm) was dropwise added into the solution until the color changed from light brown to black. The black solution was treated in a dialysis bag (100 Da) for 24 h, which was then lyophilized to obtain black solid powder, namely CDs@Pt SAs/NCs.

### Loading of DOX and the Release Behavior of DOX from CDs@Pt SAs/NCs@DOX

DOX (4 mL, 1.0 mg mL^−1^) was gradually added into CDs@Pt SAs/NCs (6 mL, 3.33 mg mL^−1^) under magnetic agitation (500 rpm) and reacted in the dark for 24 h. The solution was purified by dialysis (100–500 Da) for 6 h and lyophilized to produce dark red CDs@Pt SAs/NCs@DOX solid powder. The drug loading capacity (LC) was calculated via the absorbance at 480 nm of DOX by the following equation:

(1) $$\mathrm{LC}\left(\%\right)=\frac{mass\;of\;the\;encapsulated\;DOX}{total\;mass\;of\;the\;nano-assembly}\times 100\%$$

The drug release behavior in vitro was performed as follows. CDs@Pt SAs/NCs@DOX (3.0 mg) was dissolved in PBS solution (3.0 mL) with different pHs (6.5, 7.0, and 7.4) to dialyze in a dialysis bag (100–500 Da) against different pHs at room temperature. The absorbance at 480 nm was measured by siphoning solution (20 µL) from the dialysis bag at regular intervals to calculate the release of DOX. The CDs@Pt SAs/NCs@DOX aqueous solution (pH 7.0) irradiated by an 808 nm (0.37 W cm^−2^) laser for 8 min was used to study the DOX release behavior. The irradiation time was controlled by tuning on or off the laser during the whole study.

### POD‐ and GSHOx‐Like Activities of CDs@Pt SAs/NCs and CDs@Pt SAs/NCs@DOX

Using 3,3ʹ,5,5ʹ‐tetramethylbenzidine (TMB) as substrate, POD‐like activities of CDs@Pt SAs/NCs and CDs@Pt SAs/NCs@DOX were measured. Some factors (temperature, react time, pH, and concentration of CDs@Pt SAs/NCs or CDs@Pt SAs/NCs@DOX) that may affect the POD‐like activity of CDs@Pt SAs/NCs and CDs@Pt SAs/NCs@DOX were explored in detail.

By fitting the reaction velocity values and the substrate concentrations, the kinetics constants *K*_m_ and *V*_max_ can be calculated by the following Michaelis‐Menten equation:^[^ ^4^ ^]^

(2) $$V\;=\;\left(V_{max}\times \;C\right)/\left(K_{m}+C\right)$$

where *V* and *V*_max_ are the initial reaction velocity and the maximal reaction rate at which a nanozyme is saturated with a substrate, respectively. *C* and *K*_m_ are the substrate concentration and the Michaelis–Menten constant. *K*_m_ is an important indicator reflexing the affinity between the nano‐enzyme for its substrate, which is defined as the substrate concentration at half the maximum velocity (*V*_max_).

GSHOx‐like activities of CDs@Pt SAs/NCs and CDs@Pt SAs/NCs@DOX were investigated via fluorescence and UV–vis absorption spectra. To obtain the optimum reaction time, the mixture of CDs@Pt SAs/NCs (0.5 mg mL^−1^) and GSH (1.0 mm) was incubated at 37 °C for different times to test the fluorescence intensity. The fluorescence intensities of CDs@Pt SAs/NCs (0.5 mg mL^−1^) with different concentrations of GSH were measured after incubated at 37 °C for 60 min to study the GSHOx‐like activity. The GSHOx‐like activity of CDs@Pt SAs/NCs@DOX was studied in the same way as that of CDs@Pt SAs/NCs. Steady‐state kinetic assays were performed in PBS solution (pH 4.0) with CDs@Pt SAs/NCs as catalysts in the presence of DTNB (1.6 mg mL^−1^) and various concentrations of GSH (0.6, 0.7, 0.9, 1.0, 1.3, and 1.6 mm). The *K*_m_ and *V*_max_ were calculated according to Equation (2).

### ^1^O_2_ Detection

ESR was used for the detection of ^1^O_2_. CDs@Pt SAs/NCs or CDs@Pt SAs/NCs@DOX was mixed with H_2_O_2_ for 3 min. Then, TEMP was used as the spin‐trapping agent for ^1^O_2_ detection. The final concentrations of TEMP, H_2_O_2_, CDs@Pt SAs/NCs, and CDs@Pt SAs/NCs@DOX were 2.9 m; 25.0 mm; 250.0 and 250.0 µg mL^−1^, respectively.

### Photothermal Effect of CDs@Pt SAs/NCs and CDs@Pt SAs/NCs@DOX

The temperature changes of CDs@Pt SAs/NCs with different concentrations (50, 100, 200, and 500 µg mL^−1^) under the laser (808 nm) irradiation at the powder density of 0.37 W cm^−2^ were collected by the IR thermal camera. Photostability of CDs@Pt SAs/NCs (200 µg mL^−1^) under 808 nm laser irradiation was conducted for six cycles.

The photothermal conversion efficiency (*η*) was calculated according to the following formula:

(3) $$\eta =\frac{hA\left(\Delta T_{\max,\mathrm{mix}}-\Delta T_{\max,\mathrm{water}}\right)}{I\left(1-10^{-A_{\lambda }}\right)}$$

where *h* and *A* are the heat transfer coefficient and the surface area of the container, respectively. *ΔT*_max,mix_ and *ΔT*_max,water_ are the temperature changes of CDs@Pt SAs/NCs solution and water at the maximum steady‐state temperature, respectively. *I* and *A*_λ_ are the laser power and the absorbance of CDs@Pt SAs/NCs solution at 808 nm, respectively.

*h* and *A* can be gotten by the following equation:

(4) $$t\;=\;-\;\frac{\sum_{i}m_{i}C_{p,i}}{hA}\ln\theta$$

where *m* and *C*_p_ are the mass and heat capacity of solution (water), respectively. *Σ_i_m*_i_ *C*_p_ *_,_*_i_/*hA* can be calculated from a linear relationship between t (the time from the cooling period) and ‐ln(*θ*) (*θ* is defined as the ratio of Δ*T* to Δ*T*_max_). The masses of water and material were 2 × 10^−3^ and 2 × 10^−7^ kg, respectively, and the mass of material was much smaller than that of water. Generally, the specific heat of water (4.2 × 10^−3^ J (kg × °C)^−1^) is much higher than other materials, so *m*_material_ and *C*_p, material_ of other materials can be neglected. Finally, *hA* could be calculated by the simplified equation.

The temperature changes (*Δ* *T*) of CDs@Pt SAs/NCs dispersions during 400 s of 808 nm laser irradiation and 410 s of laser shutdown were recorded in detail. The photothermal conversion efficiency (*η*) of CDs@Pt SAs/NCs (200 µg mL^−1^) was calculated according to Equation (4) and Eqaution (5). *∆T*_max,mix_ and *∆T*_max,water_ were calculated as 14.3 and 4.1 °C, respectively. *I* and *A*_λ_ of CDs@Pt SAs/NCs at 808 nm were 0.37 W cm^−2^ and 0.2, respectively. The *η* value of CDs@Pt SAs/NCs (200 µg mL^−1^) was calculated to be ≈7.73%. The study process of the photothermal effect of CDs@Pt SAs/NCs@DOX was the same as that of CDs@Pt SAs/NCs.

### Cell Culture

Free DMEM medium containing 10% FBS, 100 U mL^−1^ penicillin, and 100 µg mL^−1^ streptomycin was used to harvest human cervical cancer (HeLa) cells at 37 °C with 5% CO_2_ in the air. Free 1640 medium containing 10% FBS, 100 U mL^−1^ penicillin, and 100 µg mL^−1^ streptomycin as cell culture medium, human normal liver (LO2) and murine mammary carcinoma (4T1) cells were harvested at 37 °C with 5% CO_2_ in the air.

### Cytotoxicity of CDs@Pt SAs/NCs, CDs@Pt SAs/NCs@DOX, and DOX

The MTT method was applied to explore the cytotoxicity of CDs@Pt SAs/NCs, CDs@Pt SAs/NCs@DOX, and DOX. In brief, LO2, 4T1, and HeLa cells were seeded in the 96‐well plates at 37 °C with 5% CO_2_ in air for 24 h. No cells were inoculated into the outermost circle of the 96‐well plate, serving as a plate blank. In each group of six parallel cells, the cells were treated with different concentrations of DOX, CDs@Pt SAs/NCs, CDs@Pt SAs/NCs + laser, CDs@Pt SAs/NCs@DOX, and CDs@Pt SAs/NCs@DOX + laser for 12 h, respectively. And then some groups were irradiated by 808 nm laser (1.0 W cm^−2^) for 5 min and continued to incubate for another 12 h. All culture media were removed and replaced by fresh culture media containing 0.5 mg mL^−1^ MTT (100 µL) to the plates in the incubator for 4 h. Finally, the optical density (OD) of the solution was measured at 570 nm via a microplate reader (Tecan, USA). DOX and CDs@Pt SAs/NCs@DOX were at an equivalent DOX concentration of 1, 2, 5, 10, and 20 µg mL^−1^. Based on the loading of DOX in CDs@Pt SAs/NCs@DOX, the corresponding Pt content in CDs@Pt SAs/NCs@DOX was found, and finally the concentration of CDs@Pt SAs/NCs was obtained. All concentrations of CDs@Pt SAs/NCs@DOX and CDs@Pt SAs/NCs were converted uniformly to DOX equivalent concentrations. The concentrations of CDs@Pt SAs/NCs (6.4, 13, 32.4, 64.8, and 130 µg mL^−1^) were calculated from the Pt‐equivalent concentrations of CDs@Pt SAs/NCs@DOX (7.5, 15, 37.4, 74.8, and 150 µg mL^−1^). The survival rate of the cells was calculated according to the following formula:

(5) $$\mathrm{Cell}\;\mathrm{viability}\;\left(\%\right)\;=\;\frac{OD_{\mathrm{Treated}}-OD_{\mathrm{Blank}}}{OD_{\mathrm{Control}}-\;OD_{\mathrm{Blank}}}\;\times \;100\%$$

where *OD*_Treated_ and *OD*_Control_ are the measured *OD* values of the cells treated with and without different concentrations of the above materials, respectively. *OD*_Blank_ is the *OD* value of the plate blank without cells.

### Calcein‐AM/PI Staining Assay

HeLa cells were inoculated in the confocal laser dishes and incubated at 37 °C with 5% CO_2_ in air for 24 h for adherence. After the cells were respectively treated with DPBS, DOX, CDs@Pt SAs/NCs, CDs@Pt SAs/NCs + laser, CDs@Pt SAs/NCs@DOX, and CDs@Pt SAs/NCs@DOX + laser for 12 h, the cells requiring laser irradiation were treated with 808 nm laser (1.0 W cm^−2^) for 5 min, and then all cells were placed in an incubator for further incubation for another 12 h. Discard all culture medium and continue incubating the cells with DPBS containing 2.0 µM Calcein‐AM and 4.5 µM PI for 15 min. Finally, after the cells were washed three times by DPBS, 0.5 mL DPBS were retained in the laser confocal dishes for imaging of living and dead cells under a laser scanning confocal microscope (LSCM, ZEISS LSM 710, Germany).

### Cellular Uptake Assays of CDs@Pt SAs/NCs and CDs@Pt SAs/NCs@DOX

HeLa cells with a density of 2 × 10^6^ cells/dish were incubated at 37 °C with 5% CO_2_ in air for 24 h for adhesion. The cells were treated with DPBS and different concentrations of CDs@Pt SAs/NCs and CDs@Pt SAs/NCs@DOX for 3 h, respectively, and then washed twice with DPBS. Cells obtained after trypsin digestion were heated with Lefort aqua regia (HNO_3_:HCl = 3:1) at 100 °C for 2 h to dissolve intracellular CDs@Pt SAs/NCs and CDs@Pt SAs/NCs@DOX. The Pt content of the obtained solutions was analyzed by ICP‐MS (Agilent 8900, USA). The concentrations of CDs@Pt SAs/NCs@DOX and CDs@Pt SAs/NCs were calculated to be 0 and 0 µg mL^−1^, 74.8 and 64.8 µg mL^−1^, and 150 and 130 µg mL^−1^, respectively, when the concentration of DOX was fixed at 0, 10, and 20 µg mL^−1^.

### In Vitro Permeability Study of 4T1 Multicellular Spheroids (MCSs)

The permeabilities of DOX, CDs@Pt SAs/NCs, and CDs@Pt SAs/NCs@DOX in the 4T1 MCSs were studied in detail. 1500 4T1 Cells/well were inoculated on the 96‐well plates and incubated for 10 days to form the MCSs. During the culture process, the plates could not be moved significantly, and the media should be changed slowly. After the 4T1 MCSs were successfully constructed, fresh culture media containing DOX (10 µg mL^−1^), CDs@Pt SAs/NCs (65 µg mL^−1^), or CDs@Pt SAs/NCs@DOX (75 µg mL^−1^) were used to handle the MCSs, respectively. The MCSs were incubated at 37 °C with 5% CO_2_ in the air for 24 h and then imaged under an LSCM (ZEISS LSM 710, Germany).

### Mice and Ethics Statement

Male BALB/c mice (5 weeks old) were purchased from SPF Biotechnology Co., Ltd. (Beijing, China). All BALB/c mice were maintained in specific pathogen‐free conditions according to the Guidelines for the Care and Use of Experimental Animals and Animal Use established by the National Institutes of Health (NIH). All related experiments were viewed and approved by the Committee of Scientific Research at Shanxi University with approval No. SXULL2021024.

### Biosafety Studies

Twelve BALB/c mice were randomly divided into four groups and treated with 100 µL DPBS or different concentrations of CDs@Pt SAs/NCs@DOX (equivalent DOX concentration: 1, 5, and 7 mg kg^−1^) every three days, respectively. After 15 days of treatment, the blood was collected from orbits for blood routine analysis and blood biochemical analysis. The blood routine analysis included the percentage of lymph cells (Lymph%), percentage of monocytes (Mon%), percentage of neutrophile granulocyte (Gran%), white blood cells (WBC), red blood cells (RBC), hemoglobin (HGB), hematocrit (HCT), mean corpuscular volume (MCV), mean content of hemoglobin (MCH), mean corpuscular‐hemoglobin concentration (MCHC), platelet (PLT), and mean platelet volume (MPV) were measured with full blood. The blood biochemical analysis of hepatic function biomarkers (ALT: alanine transferase; AST: aspartate aminotransferase) and kidney function biomarkers (UREA: urea; UA: uric acid) were estimated with the blood serum. The main organs (heart, liver, spleen, lung, and kidney) were collected to perform hematoxylin and eosin (H&E) assay.

### Blood Circulation and Tissue Distributions

The 4T1 tumor‐bearing mice model was established by subcutaneously injecting 4T1 cells (100 µL, 2.6 × 10^7^ cells mL^−1^) suspended in DPBS into the right upper extremity of each BALB/c mice. About 10 days after 4T1 cells injection, mice bearing tumors of 100–200 mm^3^ were obtained for the subsequent experiments. 4T1 tumor‐bearing BALB/c mice were injected with CDs@Pt SAs/NCs or CDs@Pt SAs/NCs@DOX at Pt concentration of 2.255 mg mL^−1^, respectively. 10 µL of blood was drawn from the tail of the mice at 15 and 30 min and 1, 2, 4, 8, 12, 24, and 48 h post‐injection. The tumor and main organs (heart, liver, spleen, lung, and kidney) were excised at 1, 2, 4, 8, 12, 24, and 48 h post‐injection. The blood, tumor, and main organs were digested with Lefort aqua regia, which were used to detect Pt content by ICP‐OES.

### Ex Vivo Fluorescence Images of CDs@Pt SAs/NCs and CDs@Pt SAs/NCs@DOX

4T1 tumor‐bearing mice were injected with CDs@Pt SAs/NCs or CDs@Pt SAs/NCs@DOX at Pt concentration of 2.255 mg mL^−1^, respectively, which were euthanized to get main organs (heart, liver, spleen, lung, and kidney) as well as tumors at 2, 4, 8, 12, 24, and 48 h post‐injection. The collected organs and tumors were imaged by an In Vivo Fx Pro living imaging system (Bruker, USA). CDs emit blue fluorescence and can be used to capture fluorescent signals from isolated tumors and major organs.

### In Vivo Tumor Suppression Studies

4T1 tumor‐bearing mice were randomly divided into eight groups (*n* = 5), which were treated with (G1) DPBS, (G2) DPBS + laser, (G3) DOX, (G4) DOX + laser, (G5) CDs@Pt SAs/NCs, (G6) CDs@Pt SAs/NCs + laser, (G7) CDs@Pt SAs/NCs@DOX, and (G8) CDs@Pt SAs/NCs@DOX + laser at DOX concentration of 5 mg kg^−1^ or Pt concentration of 2.255 mg mL^−1^, respectively, in day 0, 3, 6, 9, and 12. The tumor volume and body weight of mice in each group was monitored every 3 days on the 15 days of treatment. Subcutaneous tumor long diameter (*a* mm) and short diameter (*b* mm) were measured every 3 days to calculate the tumor volume (V = 1/2*ab* ^2^ mm^3^). For groups G2, G4, G6, and G8, the mice were irradiated by 808 nm laser (1.0 W cm^−2^) for 15 min at 1 h post‐injection. During the irradiation process, the temperature changes were monitored by the infrared thermal camera. Due to the rapid growth of malignant 4T1 tumor, the tumor in some individuals in G1 group reached 2000 mm^3^ in 15 days. The mice were marked as dead when the tumor size reached 2000 mm^3^.^[^ ^40^ ^]^ The mice were euthanized to obtain the blood, tumors, and main organs for further experiments. The blood was applied for blood routine analysis and blood biochemical analysis. Tumors and organs were weighted and fixed with 4% paraformaldehyde for H&E analysis. Organ index (in *g/g*) was calculated from the ratio of the wet weight of the individual organ to whole body weight. The tumor in different groups was collected and analyzed by enzyme‐linked immunosorbent assay (ELISA) to determine the tumor necrosis factor (TNF)‐α by the cytometric bead array (CBA) kit. The tumor growth inhibition (TGI) rate was calculated according to the following equation:

(6) $$\mathrm{TGI}\;=\;\frac{V_{C}-V_{T}}{V_{C}}\;\times \;100\%$$

where *V*_C_ and *V*_T_ are the tumor volumes of the control and treatment groups, respectively.

### Statistical Analysis

All the data were presented as mean ± SD (standard deviation). One‐way ANOVA method was adopted to assess the differences between groups by using SPSS software. Differences were considered statistically significant at *p* < 0.05 (^*^ *p* < 0.05, ^**^ *p* < 0.01, ^***^ *p* < 0.001).

## Conflict of Interest

The authors declare no conflict of interest.

## Supporting information

Supporting Information

## Data Availability

The data that support the findings of this study are available from the corresponding author upon reasonable request.
