# Supplementary material for: Drug‐Primed Self‐Assembly of Platinum‐Single‐Atom Nanozyme to Regulate Cellular Redox Homeostasis Against Cancer
Source: Adv Sci (Weinh). 2023 Sep 11;10(30):2302703. doi: 10.1002/advs.202302703 (PMC10602509; doi:10.1002/advs.202302703)
Supplement: Supplementary file 1 — Supporting Information [file ADVS-10-2302703-s001.pdf]

ADVANCED SCIENCE
Open Access

Supporting Information

for *Adv. Sci.*, DOI 10.1002/advs.202302703

Drug-Primed Self-Assembly of Platinum-Single-Atom Nanozyme to Regulate Cellular Redox Homeostasis Against Cancer

Li Zhang, Qian Dong, Yumin Hao, Zihan Wang, Wenjuan Dong, Yang Liu, Yueping Dong, Hongpeng Wu, Shaomin Shuang, Chuan Dong, Zhuo Chen* and Xiaojuan Gong**

Supporting Information

Drug-Primed Self-Assembly of Platinum-Single-Atom Nanozyme to Regulate Cellular Redox Homeostasis against Cancer

Li Zhang, Qian Dong, Yumin Hao, Zihan Wang, Wenjuan Dong, Yang Liu, Yueping Dong, Hongpeng Wu, Shaomin Shuang, Chuan Dong, Zhuo Chen,* and Xiaojuan Gong**

Experimental Section

Fluorescence quantum yield (FQY) measurements: The FQY of CDs@Pt SAs/NCs was obtained by the comparative method, which is calculated using the slope of the line relation between the integrated fluorescence intensities and absorbance. In this case, FQY can be calculated using the following equation:

$$FQY = FQY_R \left\{\frac{m}{m_R}\right\} \left\{\frac{n^2}{n_R^2}\right\} \qquad (1)$$

where *m* and *n* are the slope of the linearity obtained from the plot of the absorbance against the integrated fluorescence intensities and the refractive index of solvent, respectively. *R* represents the reference fluorophore of known FQY. Fluorescein with FQY of 79% in 0.1 M NaOH was chosen as the reference. The refractive indexes of 0.1 M NaOH and ultrapure water are 1.33, (n^2/n_R^2) will be 1, so the FQY can be obtained from the quotient of the two slopes.

(a) (b)

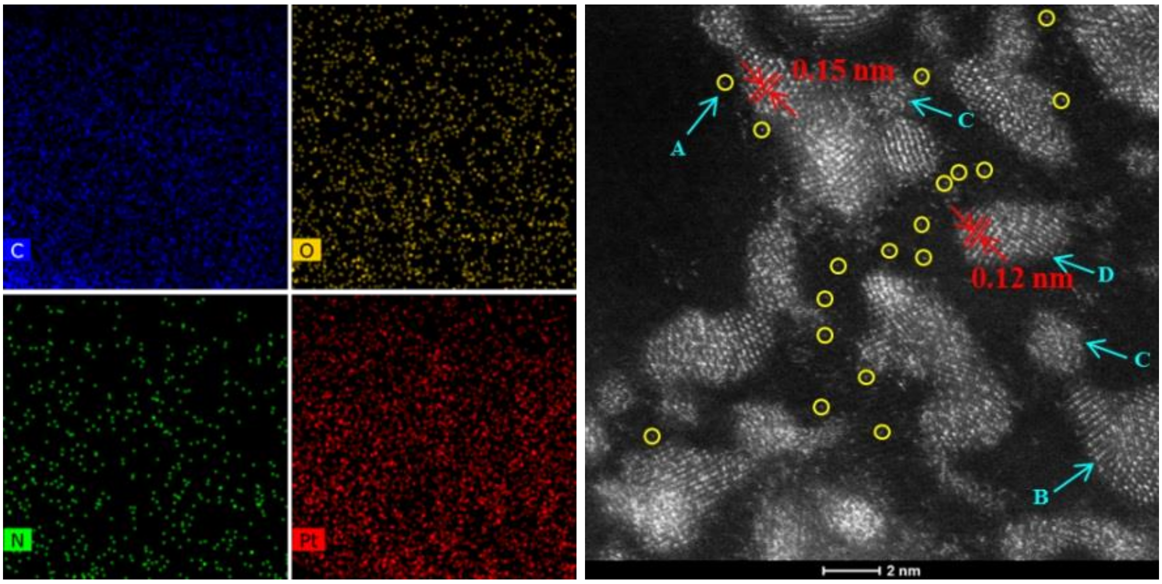

Figure S1. Characterization of CDs@Pt SAs/NCs. a) Element mapping and b) high-angle annular dark-field scanning transmission electron microscopy (HAADF-STEM) images of CDs@Pt SAs/NCs. The uniform distribution of C, O, N, and Pt elements can be found in element mapping, indicating that Pt has been successfully reduced to the surface of CDs. The HAADF-STEM image illustrates that Pt exists as single atoms (A: yellow circle) and nanoclusters (B: faced Pt clusters; C: highly disordered clusters; D: strained lattices of Pt) with the lattice fringe spacing of 0.12 nm and 0.15 nm, corresponding to the (311) and (220) plane of face-centered cubic (fcc) phase of Pt,[1,2] respectively.

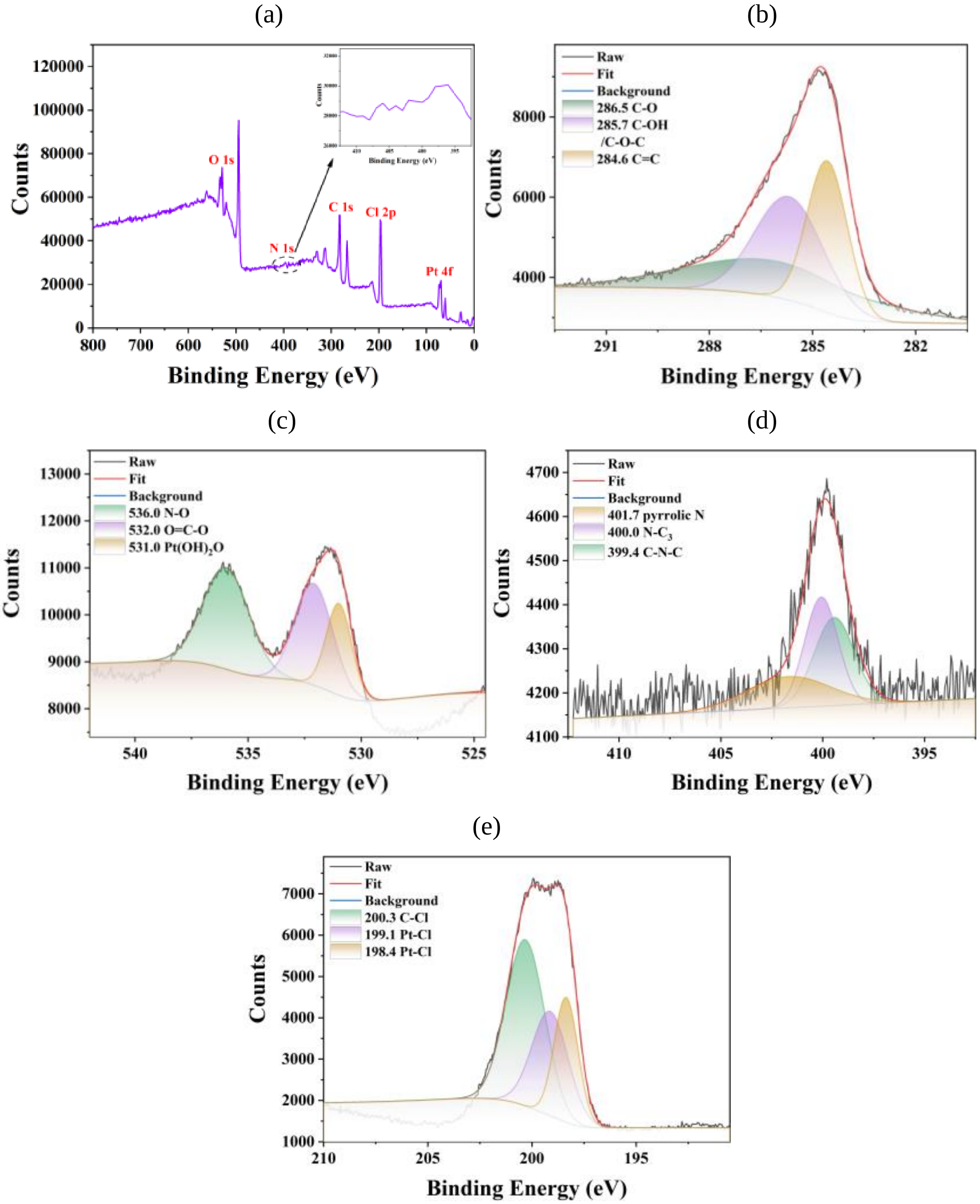

Figure S2. XPS spectra of CDs@Pt SAs/NCs. a) XPS survey scan, high-resolution b) C 1s, c) O 1s, d) N 1s, and e) Cl 2p XPS spectra of CDs@Pt SAs/NCs.

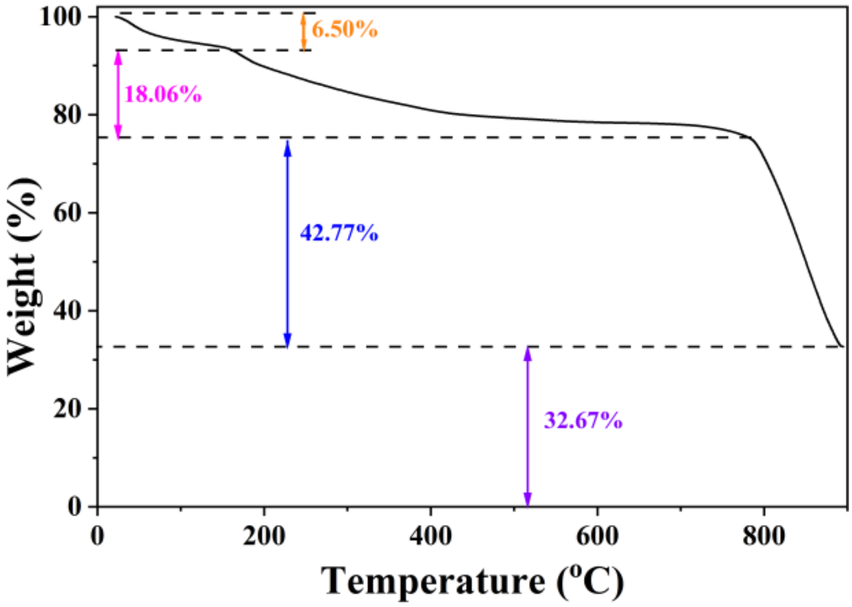

Figure S3. TGA spectrum of CDs@Pt SAs/NCs. The TGA curve was obtained by treating CDs@Pt SAs/NCs in a nitrogen (N_2) atmosphere with a heating rate of 10 ^{o}C min^{-1}. The residual weight of CDs@Pt SAs/NCs is about 32.67% when the temperature is higher than 900 ^{o}C, which is mainly inorganic Pt in CDs@Pt SAs/NCs.

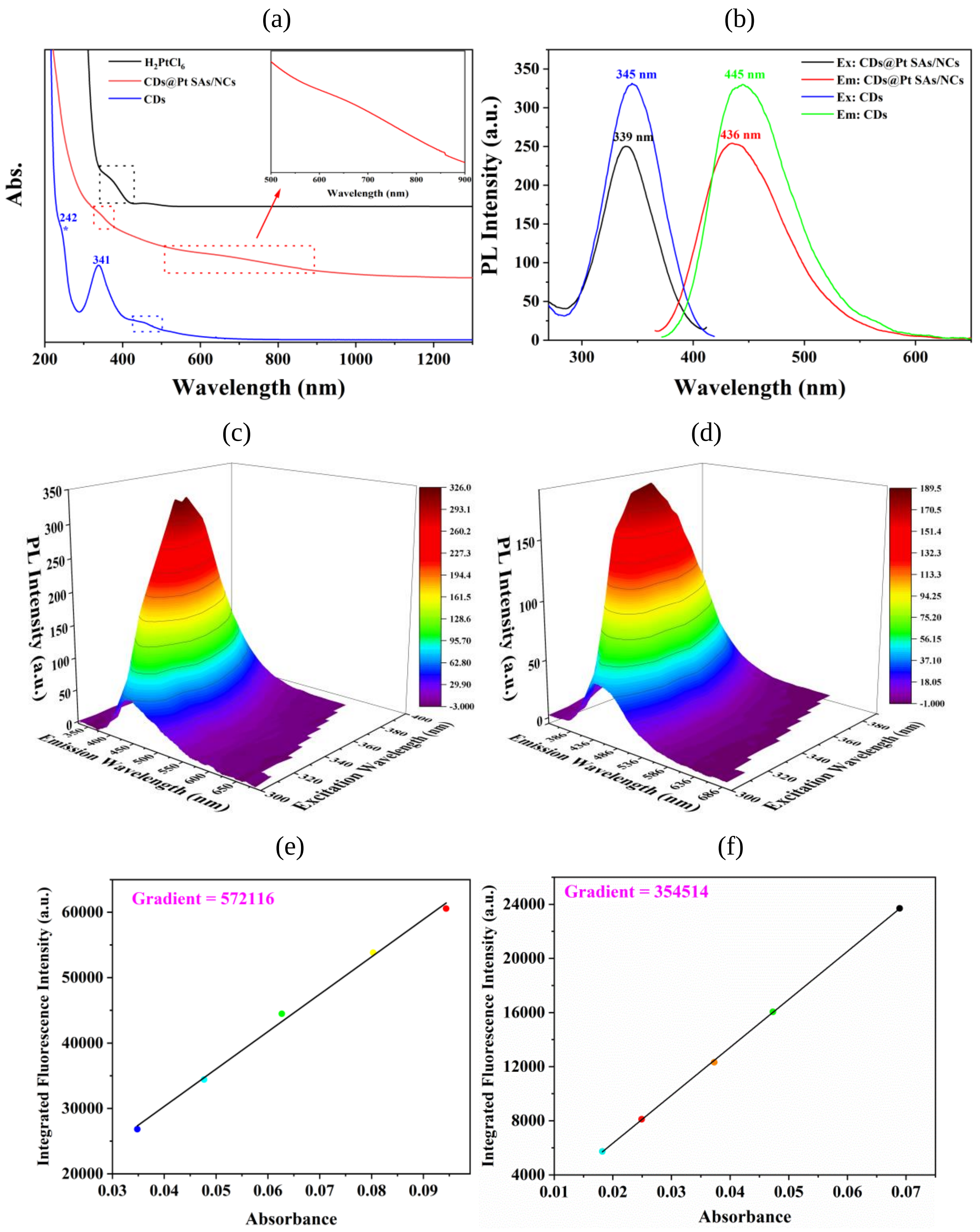
(a)
(b)
Abs.
H_2PtCl_6
CDs@Pt SAs/NCs
CDs
242
341
Wavelength (nm)
PL Intensity (a.u.)
Ex: CDs@Pt SAs/NCs
Em: CDs@Pt SAs/NCs
Ex: CDs
Em: CDs
345 nm
445 nm
339 nm
436 nm
(c)
(d)
Emission Wavelength (nm)
Excitation Wavelength (nm)
(e)
(f)
Gradient = 572116
Gradient = 354514
Integrated Fluorescence Intensity (a.u.)
Absorbance

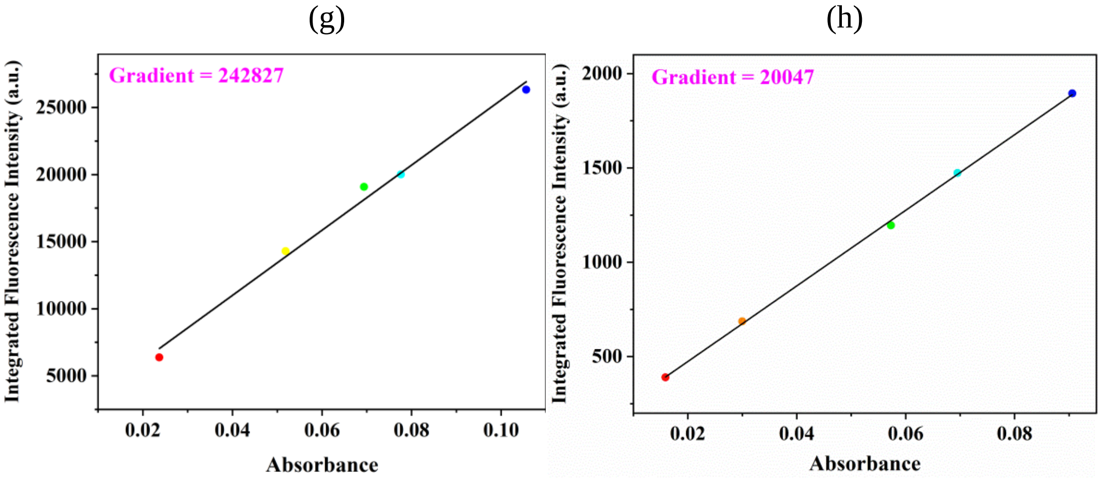

Figure S4. Characterization of CDs@Pt SAs/NCs. a) UV-vis spectra of H_2PtCl_6, CDs, and CDs@Pt SAs/NCs, inset: local enlargement of CDs@Pt SAs/NCs at 500-900 nm. b) The excitation and emission spectra of the CDs and CDs@Pt SAs/NCs. The emission spectra of c) CDs and d) CDs@Pt SAs/NCs at different λ_{ex}. Plots of integrated fluorescence intensity against absorbance of e) quinine sulfate and f) CDs at excitation and absorption wavelengths of 345 nm. Plots of integrated fluorescence intensity against absorbance of g) quinine sulfate and h) CDs@Pt SAs/NCs at excitation and absorption wavelengths of 339 nm. CDs@Pt SAs/NCs has a weak absorption peak at 324-377 nm that is obviously different from that of CDs and H_2PtCl_6, which may be a new absorption peak produced after the reaction of CDs and H_2PtCl_6. A new wide absorption peak at 500-900 nm can be found in the absorption spectrum of CDs@Pt SAs/NCs, indicating that CDs@Pt SAs/NCs have been successfully prepared and have photothermal potential. Under different excitation wavelengths, the emission wavelengths of CDs produce a redshift from 443 nm to 472 nm, while the emission wavelengths of CDs@Pt SAs/NCs hardly change between 436 nm and 440 nm, illustrating that CDs and CDs@Pt SAs/NCs have the excitation-dependent and excitation-independent photoluminescence (PL) behaviors, respectively, which may be attributed to the change of the optical properties of CDs caused by Pt SAs/NCs. Using quinine sulfate as reference, the FQYs of CDs and CDs@Pt SAs/NCs were calculated to be 33.5% and 3.05%, respectively. The high FQY of CDs may be caused by the surface-state and abundant amino functional groups, while the low FQY of CDs@Pt SAs/NCs may be attributed to the change of the surface-state and functional groups of CDs after the reduction of Pt SAs/NCs to the surface of CDs, resulting in the significant fluorescence attenuation of CDs.

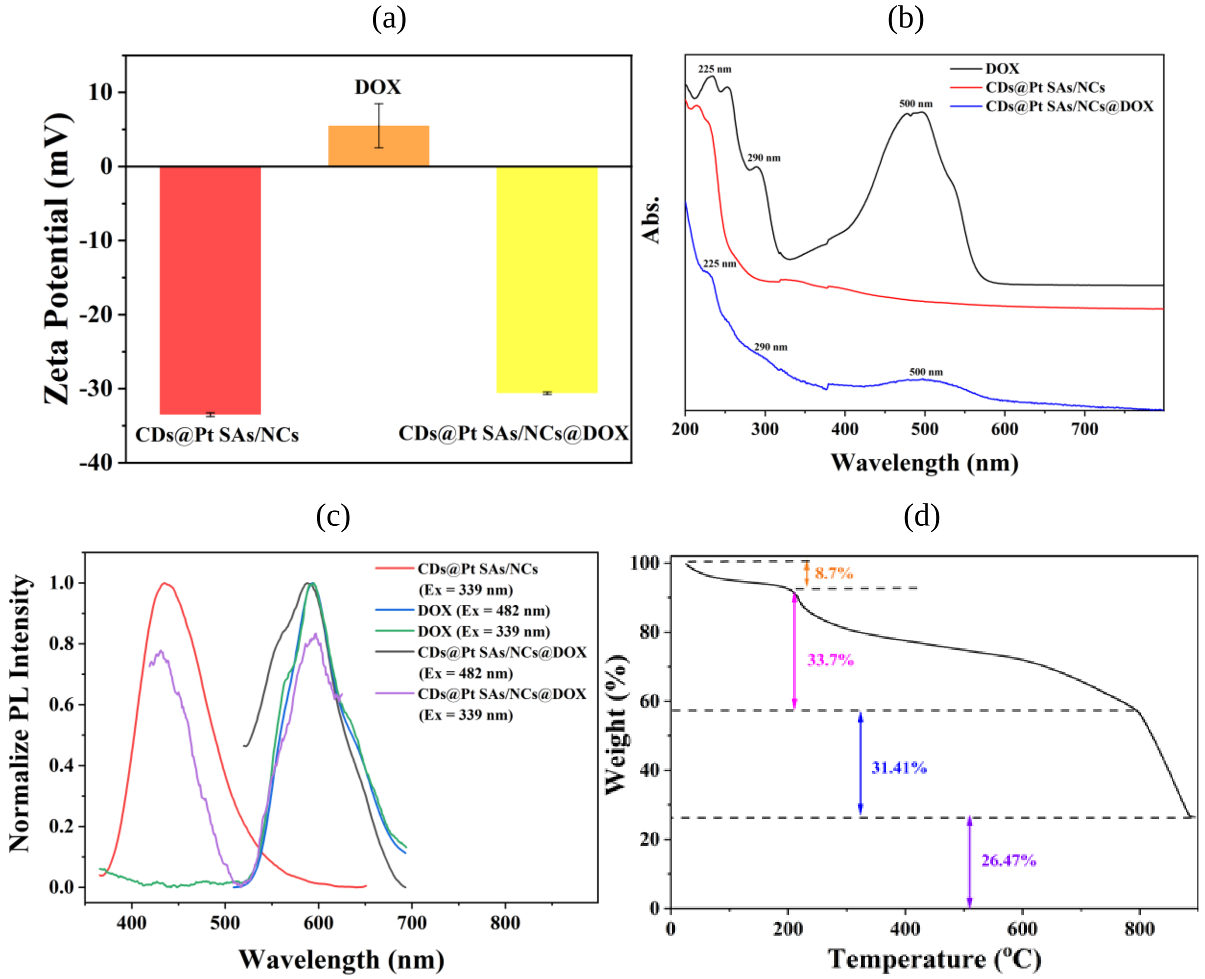

Figure S5. Characterization of CDs@Pt SAs/NCs@DOX. a) The zeta potentials and b) UV-vis absorption spectra of DOX, CDs@Pt SAs/NCs, and CDs@Pt SAs/NCs@DOX. c) The emission spectra of CDs@Pt SAs/NCs, DOX, and CDs@Pt SAs/NCs@DOX under different excitation wavelengths. d) TGA curve of CDs@Pt SAs/NCs@DOX. The characteristic absorption peaks of 225, 290, and 500 nm of DOX appeared in the absorption spectrum of CDs@Pt SAs/NCs@DOX, indicating the formation of CDs@Pt SAs/NCs@DOX and confirming the successful loading of the driving DOX molecules onto CDs@Pt SAs/NCs. Figure S5c depicts the PL spectra of CDs@Pt SAs/NCs, DOX, and CDs@Pt SAs/NCs@DOX. At an excitation wavelength of 339 nm, the emission peaks of CDs@Pt SAs/NCs and DOX were 436 and 596 nm, respectively, while the emission peaks of CDs@Pt SAs/NCs@DOX were 432 and 596 nm. At an excitation wavelength of 482 nm for CDs@Pt SAs/NCs@DOX, only the emission peak of DOX at 596 nm can be found. In essence, both CDs@Pt SAs/NCs and DOX moieties in CDs@Pt SAs/NCs@DOX can maintain their own PL characteristics.

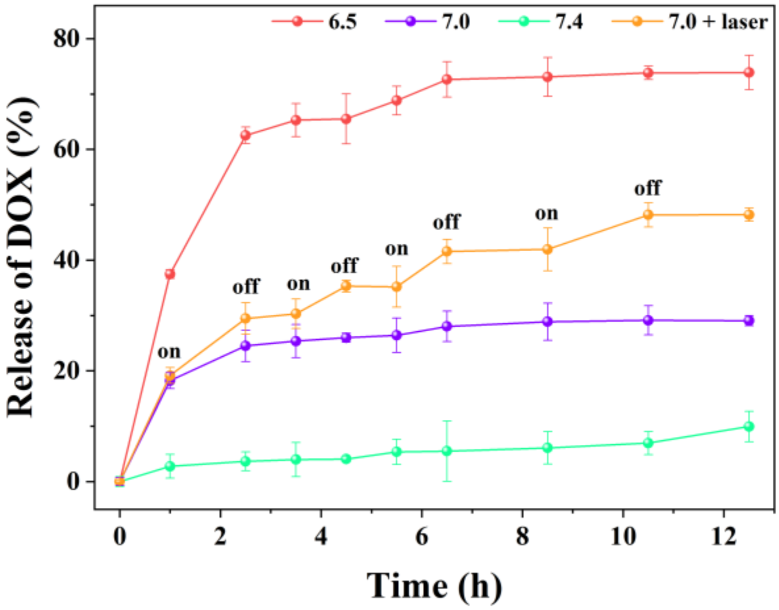

Figure S6. The DOX release behavior of CDs@Pt SAs/NCs@DOX. Effect of pH and 808 nm laser irradiation (0.37 W cm^{-2}, 8 min) on DOX release from CDs@Pt SAs/NCs@DOX (n = 3). The release of DOX in CDs@Pt SAs/NCs@DOX reached equilibrium after 6.5 h, and the release rates of DOX at pH 6.5, 7.0, and 7.4 were 74%, 29%, and 10%, respectively, suggesting that the release of DOX was controlled by pH and was much higher in a weakly acidic environment than in a neutral environment. After four cycles of 808 nm laser irradiation, the release rate of DOX increased from 29% to 48% at pH 7.0, illustrating that laser irradiation not only increased the temperature of CDs@Pt SAs/NCs@DOX, but also promoted the release of DOX from CDs@Pt SAs/NCs@DOX.

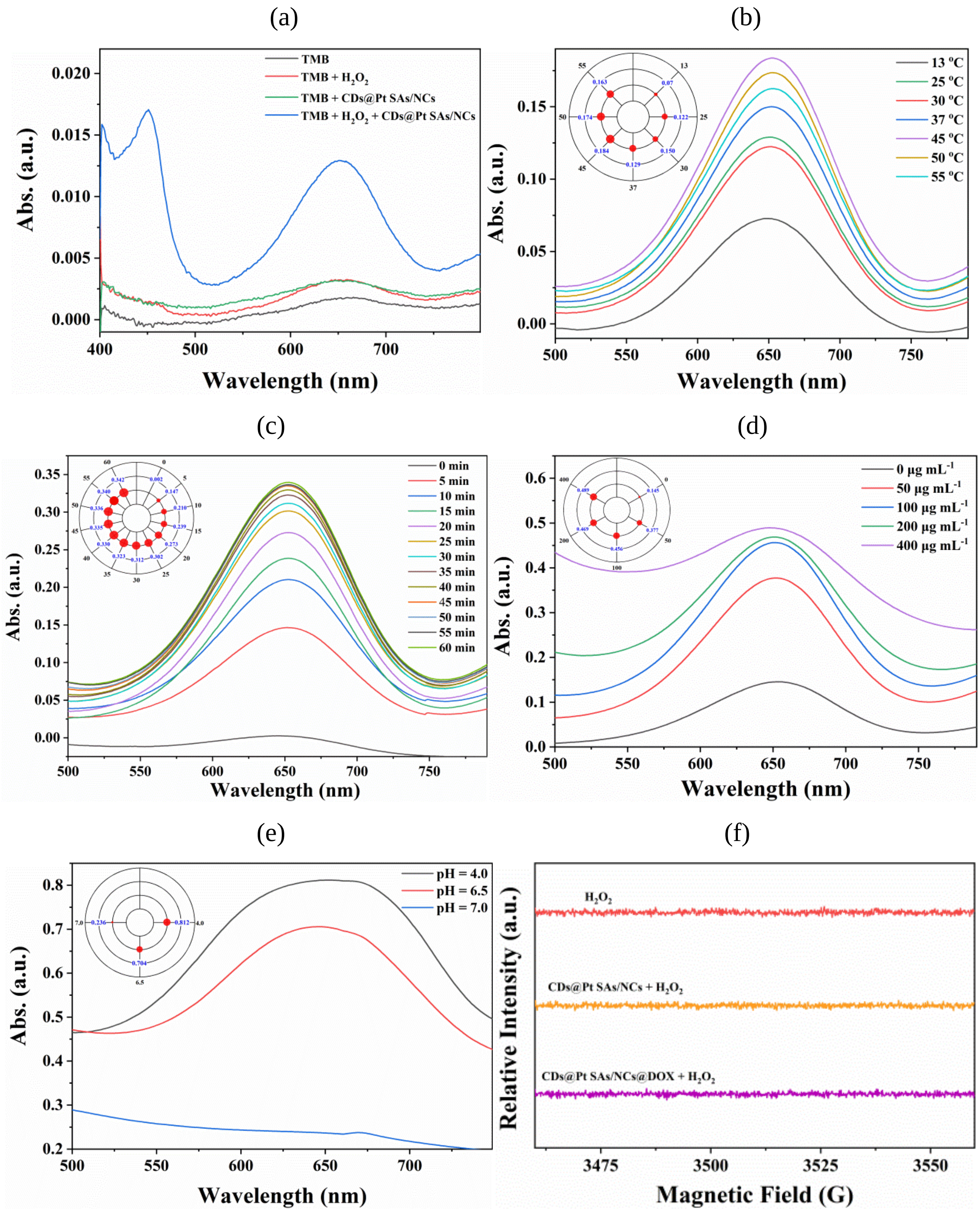

Figure S7. The POD-like performance of CDs@Pt SAs/NCs. a) Typical UV-vis absorption spectra of TMB, TMB + H_2O_2, TMB + CDs@Pt SAs/NCs, and TMB + H_2O_2 + CDs@Pt SAs/NCs. The concentrations of TMB, H_2O_2, and CDs@Pt SAs/NCs were 50 μM, 20 mM, and 1.0 μg mL^{-1}, respectively. The POD-mimic activities of CDs@Pt SAs/NCs to peroxidase substrate TMB under different conditions including b) reaction temperature, c) reaction time, d) CDs@Pt SAs/NCs concentration, and e) pH. f) ESR spectra of 5,5-dimethyl-1-pyrroline-N-oxide (DMPO)/·OH in different solutions. The POD-like activity of CDs@Pt SAs/NCs was

explored by TMB. The co-existence of TMB, H_2O_2, and CDs@Pt SAs/NCs showed an obvious absorption peak at 652 nm, indicating that CDs@Pt SAs/NCs has excellent POD-like activity. The POD-like performance follows the factors-dependent manner. The reaction temperature, reaction time, CDs@Pt SAs/NCs concentration, and pH for the highest POD-like activity were 45 ^{o}C, 60 min, 200 µg mL^{-1}, and 6.5, respectively. The characteristic ·OH (1:2:2:1) signal was not observed in the ESR spectra, indicating that CDs@Pt SAs/NCs with POD activity does not produce ·OH.

(a)

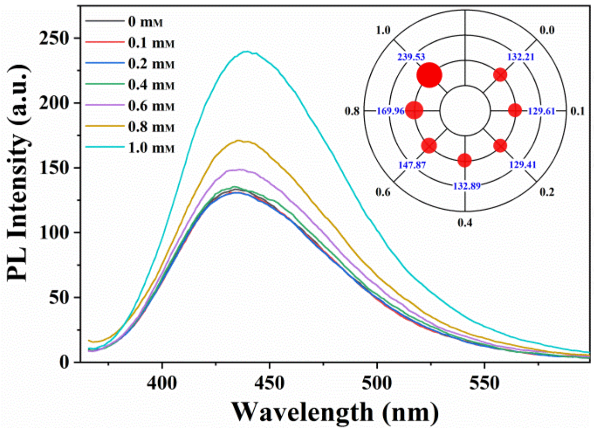
PL Intensity (a.u.)
Wavelength (nm)
0 mM
0.1 mM
0.2 mM
0.4 mM
0.6 mM
0.8 mM
1.0 mM

(b)

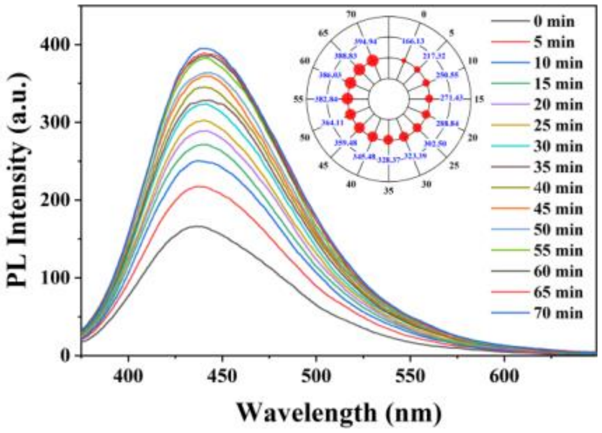
PL Intensity (a.u.)
Wavelength (nm)
0 min
5 min
10 min
15 min
20 min
25 min
30 min
35 min
40 min
45 min
50 min
55 min
60 min
65 min
70 min

(c)

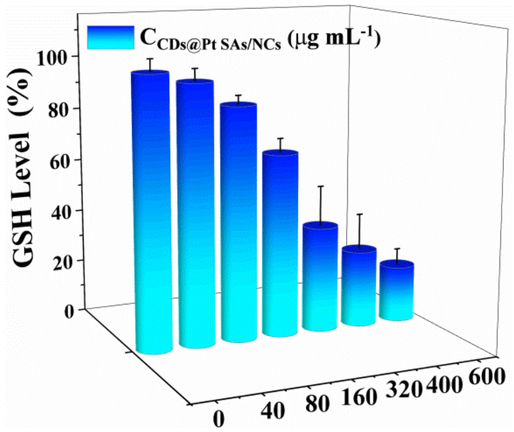
GSH Level (%)
C CDs@Pt SAs/NCs (μg mL-1)

(d)

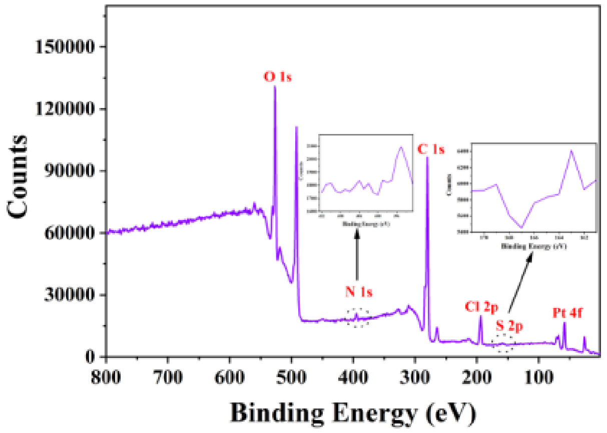
Counts
Binding Energy (eV)
O 1s
C 1s
N 1s
Cl 2p
S 2p
Pt 4f

(e)

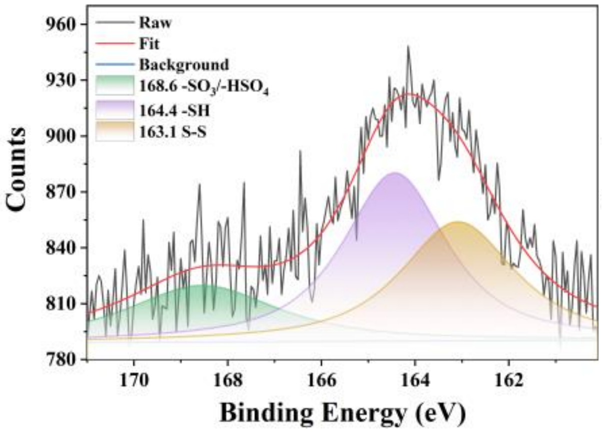
Counts
Binding Energy (eV)
Raw
Fit
Background
168.6 -SO3/-HSO4
164.4 -SH
163.1 S-S

(f)

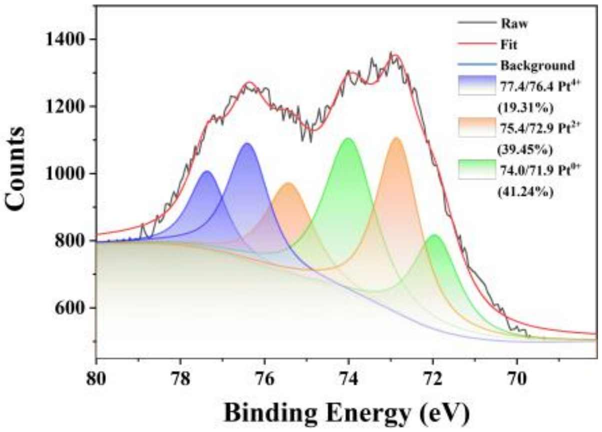
Counts
Binding Energy (eV)
Raw
Fit
Background
77.4/76.4 Pt4+
(19.31%)
75.4/72.9 Pt2+
(39.45%)
74.0/71.9 Pt0+
(41.24%)

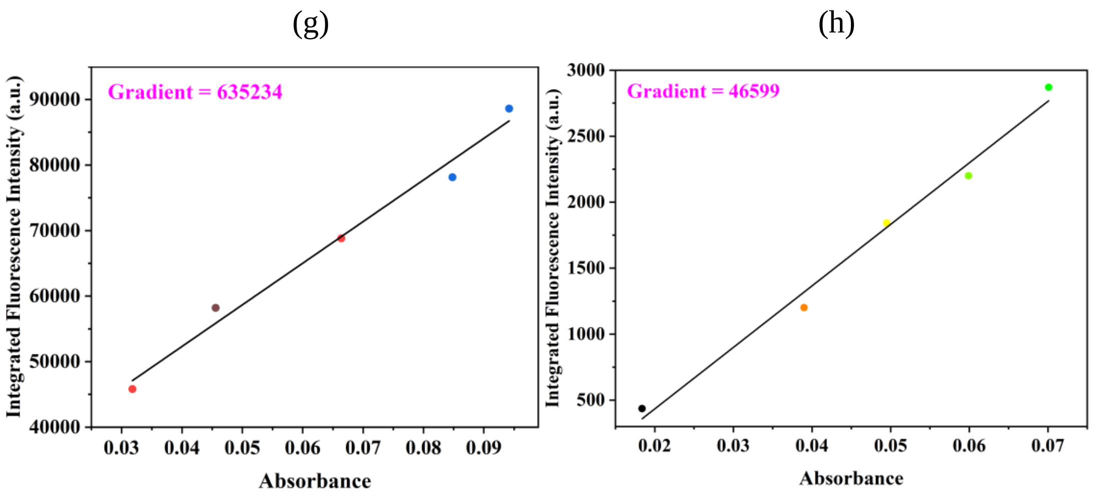

Figure S8. The GSHOx-like performance of CDs@Pt SAs/NCs. a) PL spectra of CDs@Pt SAs/NCs (0.5 mg mL^{-1}) with different concentrations of GSH (0.0-1.0 mM). b) PL spectra of CDs@Pt SAs/NCs (0.5 mg mL^{-1}) mixed with GSH (1.0 mM) for different times (0-70 min). Inset: The area of solid red dot represents the PL intensity (blue number). c) Depletion capacity of GSH at different concentrations of CDs@Pt SAs/NCs (*n* = 3). d) XPS survey scan and high-resolution e) S 2p and f) Pt 4f XPS of CDs@Pt SAs/NCs reacting with GSH. Plots of integrated fluorescence intensity against absorbance of g) quinine sulfate and h) CDs@Pt SAs/NCs reacting with GSH at excitation and absorption wavelengths of 339 nm.

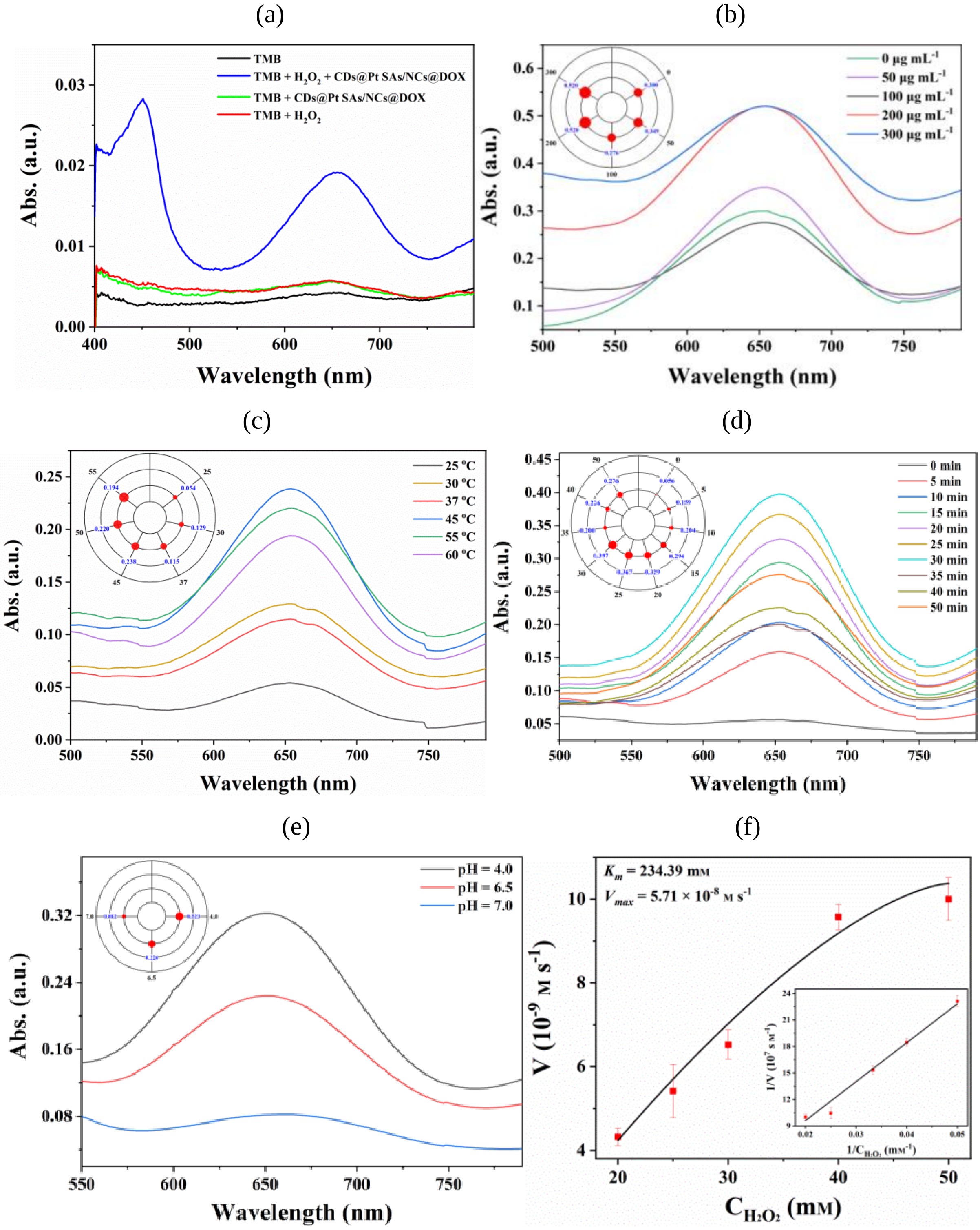

(a)
(b)
TMB
TMB + H2O2 + CDs@Pt SAs/NCs@DOX
TMB + CDs@Pt SAs/NCs@DOX
TMB + H2O2
Abs. (a.u.)
Wavelength (nm)
0 μg mL-1
50 μg mL-1
100 μg mL-1
200 μg mL-1
300 μg mL-1
(c)
(d)
25 °C
30 °C
37 °C
45 °C
55 °C
60 °C
0 min
5 min
10 min
15 min
20 min
25 min
30 min
35 min
40 min
50 min
(e)
(f)
pH = 4.0
pH = 6.5
pH = 7.0
Km = 234.39 mM
Vmax = 5.71 × 10-8 M s-1
V (10-9 M s-1)
CH2O2 (mM)
1/V (107 s M-1)
1/CH2O2 (mM-1)

(g) (h)

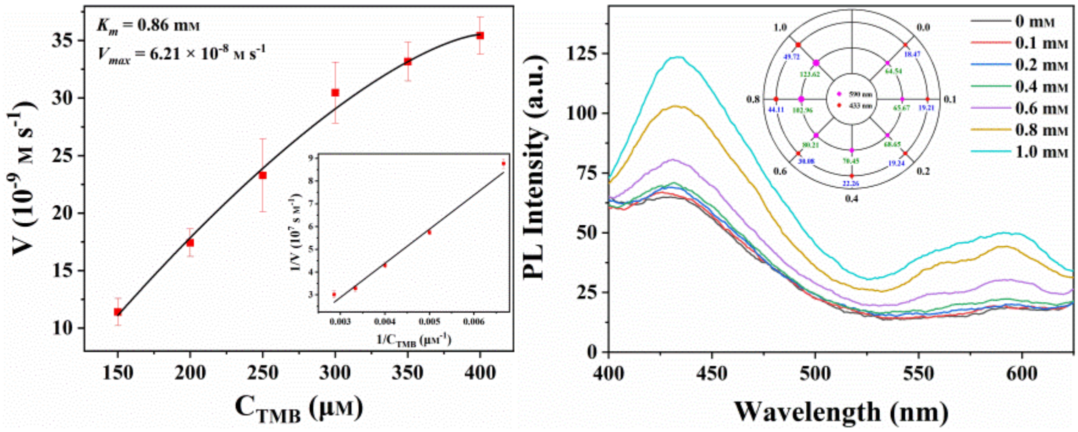

Figure S9. The POD- and GSHOx-like performances of CDs@Pt SAs/NCs@DOX. a) Typical UV-vis absorption spectra of TMB, TMB + H_2O_2, TMB + CDs@Pt SAs/NCs@DOX, and TMB + H_2O_2 + CDs@Pt SAs/NCs@DOX. The concentrations of TMB, H_2O_2, and CDs@Pt SAs/NCs@DOX are 50 μM, 20 mM, and 1.0 μg mL^{-1}, respectively. The POD-like activity of CDs@Pt SAs/NCs@DOX to peroxidase substrate TMB under different conditions including b) CDs@Pt SAs/NCs@DOX concentration, c) reaction temperature, d) reaction time, and e) pH. f) Steady-state kinetic assay and the catalytic mechanism of CDs@Pt SAs/NCs@DOX and H_2O_2 under a fixed TMB concentration of 100 μM. Inset: Double-reciprocal Lineweaver-Burk plots of catalytic activity between CDs@Pt SAs/NCs@DOX and H_2O_2 under a fixed TMB concentration. g) Steady-state kinetic assay and the catalytic mechanism of CDs@Pt SAs/NCs@DOX and TMB under a fixed H_2O_2 concentration of 20 μM. Inset: Double-reciprocal Lineweaver-Burk plots of catalytic activity between CDs@Pt SAs/NCs@DOX and TMB under a fixed H_2O_2 concentration. h) PL spectra of CDs@Pt SAs/NCs@DOX (0.5 mg mL^{-1}) with different concentrations of GSH. When the concentration of CDs@Pt SAs/NCs@DOX, reaction temperature, reaction time, and pH were 300 μg mL^{-1}, 45 °C, 30 min, and 4.0, respectively, CDs@Pt SAs/NCs@DOX can produce the most $^1O_2$ and reveal the best POD-like activity. The V_{max} of CDs@Pt SAs/NCs@DOX for H_2O_2 and TMB were 5.71×10^{-8} M s^{-1} and 6.21×10^{-8} M s^{-1}, respectively. The K_m of CDs@Pt SAs/NCs@DOX for H_2O_2 and TMB were 234.39 mM and 0.86 mM, respectively.

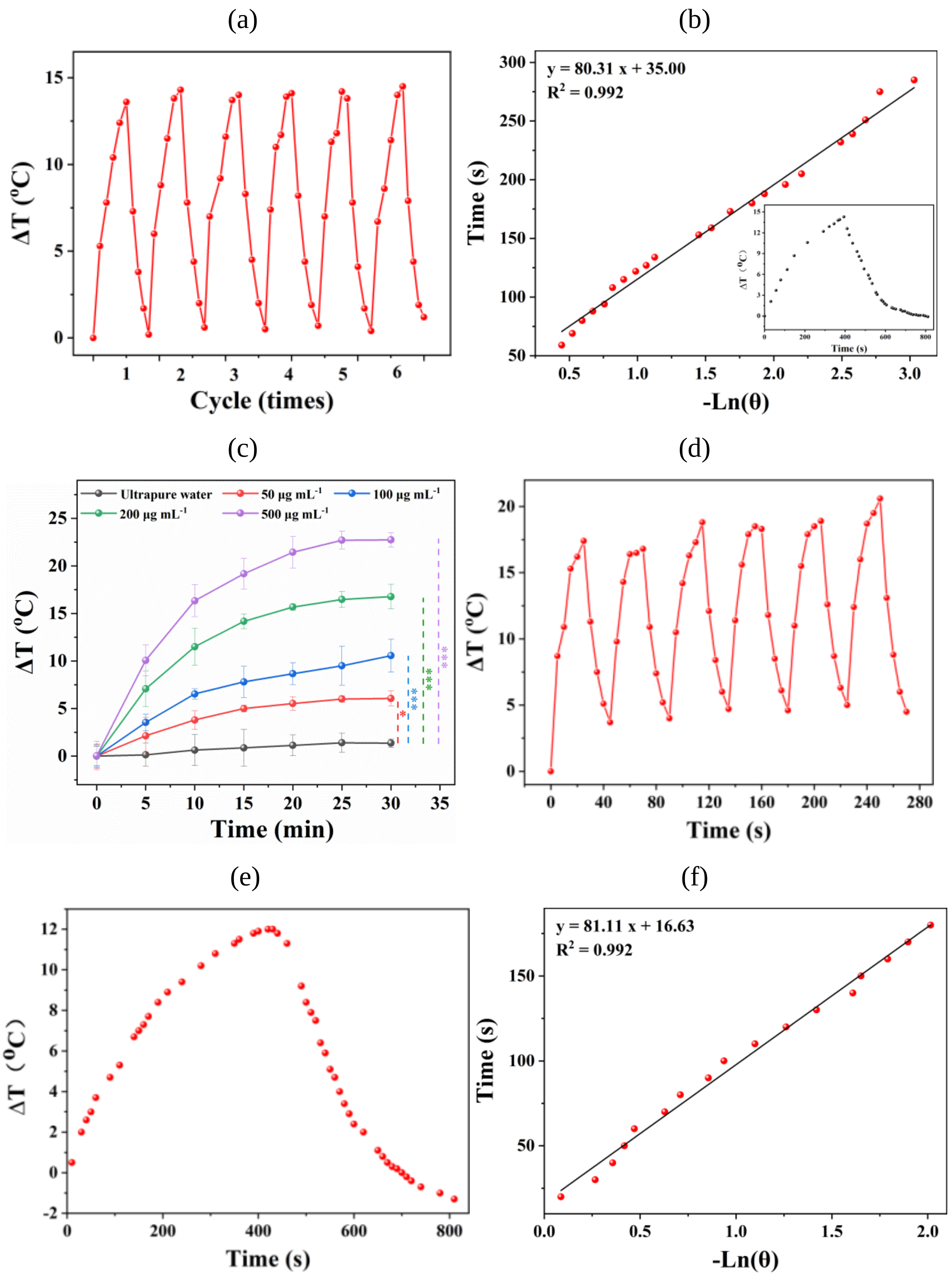

Figure S10. The photothermal effect of CDs@Pt SAs/NCs and CDs@Pt SAs/NCs@DOX. a) Temperature changes (ΔT) of CDs@Pt SAs/NCs during six on/off cycles upon the 808 nm laser irradiation. b) Linear time data obtained from the cooling period. Inset: Heating and cooling curves of CDs@Pt SAs/NCs. c) Photothermal curves of CDs@Pt SAs/NCs@DOX with various concentrations irradiated by the 808 nm laser (0.37 W cm^{-2}) at different times. ($^{*}p < 0.05$, $^{**}p < 0.01$, $^{***}p < 0.001$, $n = 3$). d) Temperature changes (ΔT) of CDs@Pt SAs/NCs@DOX during six on/off cycles upon the 808 nm laser irradiation. e) The

temperature changes (ΔT) of CDs@Pt SAs/NCs@DOX dispersion during the 808 nm laser irradiation for 400 s and the 808 nm laser shutdown for 410 s. f) Linear relationship between the time after the 808 nm laser shutdown and -ln(θ) obtained from the cooling period of Supplementary Figure S10e. CDs@Pt SAs/NCs@DOX has the same unique properties as CDs@Pt SAs/NCs, including concentration- and time-dependent photothermal effects, excellent photostability, and mild photothermal treatment effect.

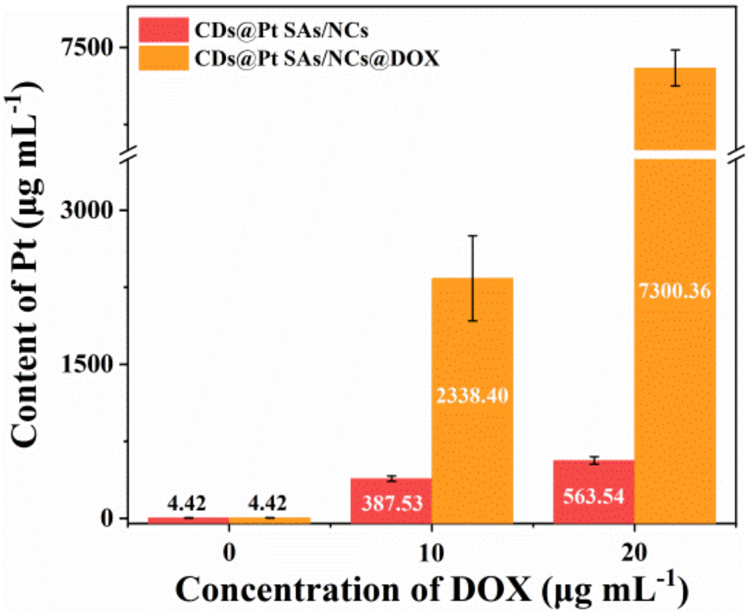

Figure S11. Uptake results of HeLa cells to different concentrations of CDs@Pt SAs/NCs and CDs@Pt SAs/NCs@DOX (n = 6).

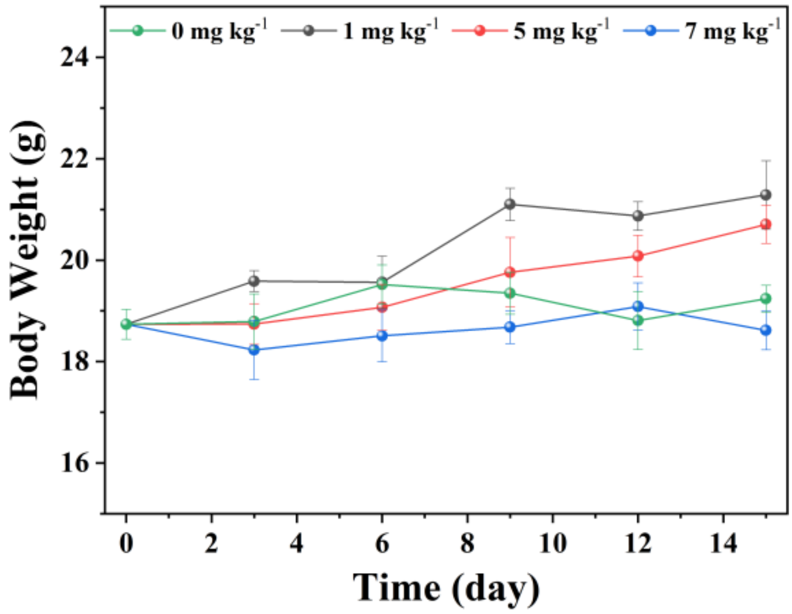

Figure S12. Changes in body weight of mice after 15 days of treatment with different concentrations of CDs@Pt SAs/NCs@DOX (0, 1, 5, and 7 mg kg^{-1}).

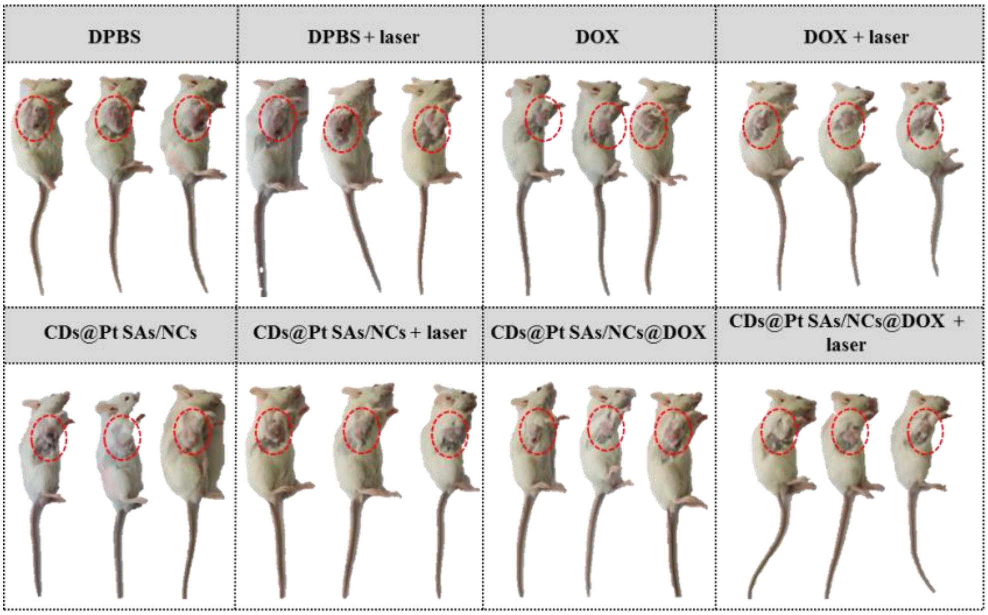

Figure S13. Typical results of the mice 25 days after inoculation.

(a)

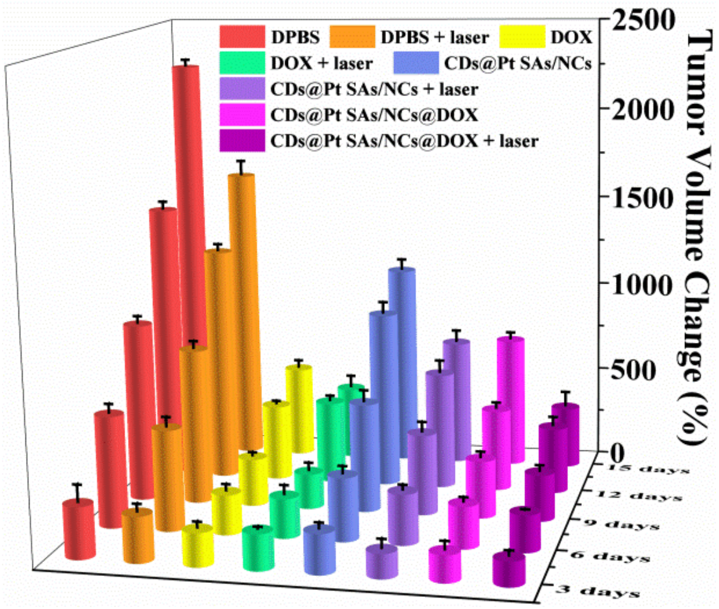

(b)

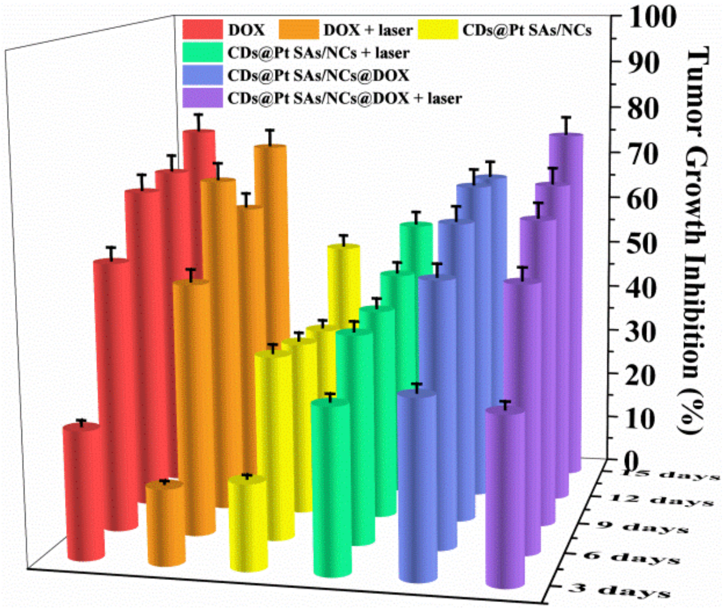

Figure S14. *In vivo* tumor suppression study. a) The tumor volume change and b) tumor growth inhibition of the mice in all groups during the treatment for 15 days.

(a)

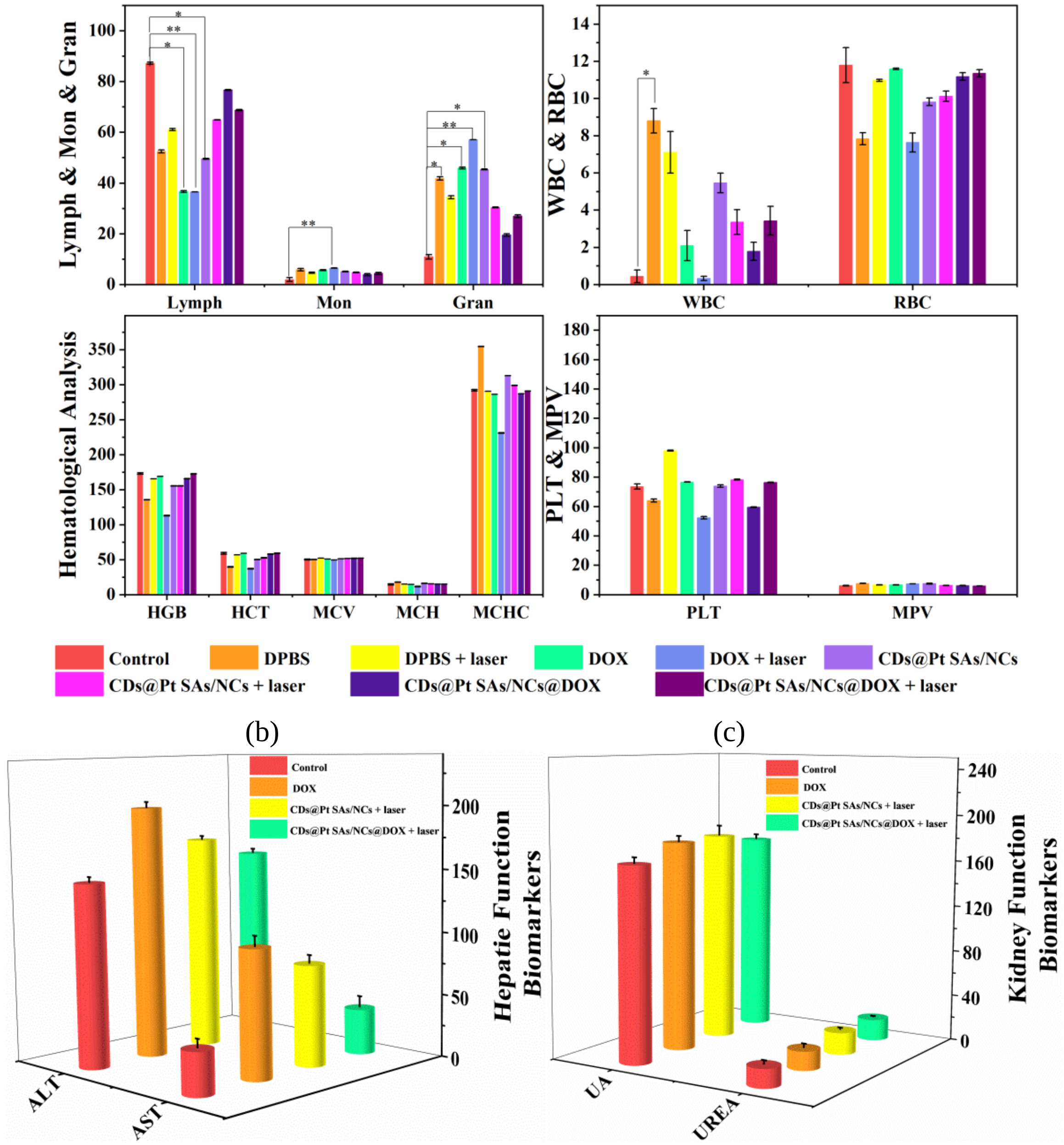

Figure S15. Hematological and blood biochemical analysis of of mice after 15 days of treatment with different treatments. a) Hematological analysis of Lymph (%), Mon (%), Gran (%), WBC (10^9 L^{-1}), RBC (10^{12} L^{-1}), HGB (g L^{-1}), HCT (%), MCV (fL), MCH (pg), MCHC (g L^{-1}), PLT (10^{10} L^{-1}), and MPV (fL). Blood biochemical analysis of b) hepatic function biomarkers (ALT, U L^{-1}; AST, U L^{-1}) and c) kidney function biomarkers (UREA, mM; UA, μM). ($^{*}p < 0.05$, $^{**}p < 0.01$, $^{***}p < 0.001$).

Table S1. The maximum reaction rate (V_{max}) and Michaelis-Menton constant (K_m) of CDs@Pt SAs/NCs, CDs@Pt SAs/NCs@DOX, natural HRP, and other SAzymes with TMB and H_2O_2 as the substrate for POD-like catalysis.

Catalyst	Substrate	V_{max} [10^{-8} M s^{-1}]	K_m [mM]	Ref.
HRP[a)]	TMB	1.41	3.759	[3]
	H_2O_2	2.76	11.63	
Fe SAEs[b)]	TMB	58.8	3.92	[4]
	H_2O_2	8.25	0.243	
PMCS[c)]	TMB	10.66	0.224	[3]
	H_2O_2	12.15	40.16	
Fe-SAzyme[d)]	TMB	18.35	0.3	[5]
	H_2O_2	9.94	0.21	
CDs@Pt SAs/NCs[e)]	TMB	12.20	0.33	This work
	H_2O_2	3.27	4.02	
CDs@Pt SAs/NCs@DOX[f)]	TMB	6.21	0.86	This work
	H_2O_2	5.71	234.39	

[a)](horseradish peroxidase); [b)](porphyrin-like single Fe sites on N-doped carbon nanomaterials); [c)](the monodispersed ZIF-8 derived carbon nanospheres containing zinc-centered porphyrin-like structure); [d)](Fe-N-C single-atom nanozyme); [e)](Pt single atoms or nanoclusters (Pt SAs/NCs) on the surface of CDs); [f)](a nano-assembly using DOX-primed CDs@Pt SAs/NCs). The V_{max} of CDs@Pt SAs/NCs@DOX for H_2O_2 and TMB were greater than HRP, indicating that CDs@Pt SAs/NCs@DOX had a faster catalytic reaction rate. The K_m of CDs@Pt SAs/NCs@DOX for H_2O_2 and TMB were higher than CDs@Pt SAs/NCs, indicating the weaker affinity between CDs@Pt SAs/NCs@DOX and substrates, which may be due to the fact that part of Pt was wrapped during the formation of CDs@Pt SAs/NCs@DOX.

Table S2. The V_{max} and K_m of CDs@Pt SAs/NCs and other nanozymes with GSH as the substrate for GSHOx-like catalysis.

Catalyst	V_{max} [10^{-6} M s^{-1}]	K_m [mM]	Ref.
Pd SAzyme[a)]	19.7	0.24	[6]
Ru SAEs[b)]	55.5	5.43	[7]
$Bi_2Fe_4O_9$ NSs[c)]	0.92	1.85	[8]
CDs@Pt SAs/NCs[d)]	7.46	1.04	This work

[a)](Single atom Pd nanozyme); [b)](Ru single-atom enzymes); [c)]($Bi_2Fe_4O_9$ nanosheets); [d)](Pt single atoms or nanoclusters (Pt SAs/NCs) on the surface of CDs).

References

[1] V. S. Ramkumar, A. Pugazhendhi, S. Prakash, N. K. Ahila, G. Vinoj, S. Selvam, G. Kumar, E. Kannapiran, R. B. Rajendran, *Biomed. Pharmacother.* **2017**, *92*, 479-490.

[2] J. Liu, *ACS Catal.* **2017**, *7*, 34-39.

[3] B. Xu, H. Wang, W. Wang, L. Gao, S. Li, X. Pan, H. Wang, H. Yang, X. Meng, Q. Wu, L. Zheng, S. Chen, X. Shi, K. Fan, X. Yan, H. Liu, *Angew. Chem. Int. Ed.* **2019**, *58*, 4911-4916.

[4] C. Zhao, C. Xiong, X. Liu, M. Qiao, Z. Li, T. Yuan, J. Wang, Y. Qu, X. Wang, F. Zhou, Q. Xu, S. Wang, M. Chen, W. Wang, Y. Li, T. Yao, Y. Wu, Y. Li, *Chem. Commun.* **2019**, *55*, 2285-2288.

[5] X. Zhou, M. Wang, J. Chen, X. Xie, X. Su, *Anal. Chim. Acta* **2020**, *1128*, 72-79.

[6] M. Chang, Z. Hou, M. Wang, C. Yang, R. Wang, F. Li, D. Liu, T. Peng, C. Li, J. Lin, *Angew. Chem. Int. Ed.* **2021**, *60*, 12971-12979.

[7] W. Wang, Y. Zhu, X. Zhu, Y. Zhao, Z. Xue, C. Xiong, Z. Wang, Y. Qu, J. Cheng, M. Chen, M. Liu, F. Zhou, H. Zhang, Z. Jiang, Y. Hu, H. Zhou, H. Wang, Y. Li, Y. Liu, Y. Wu, *ACS Appl. Mater. Interfaces* **2021**, *13*, 45269-45278.

[8] Y. Zou, B. Jin, H. Li, X. Wu, Y. Liu, H. Zhao, D. Zhong, L. Wang, W. Chen, M. Wen, Y. Liu, *ACS Nano* **2022**, *16*, 21491-21504.
